# Oligosarcomas, IDH-mutant are distinct and aggressive

**DOI:** 10.1007/s00401-021-02395-z

**Published:** 2021-12-30

**Authors:** Abigail K. Suwala, Marius Felix, Dennis Friedel, Damian Stichel, Daniel Schrimpf, Felix Hinz, Ekkehard Hewer, Leonille Schweizer, Hildegard Dohmen, Ute Pohl, Ori Staszewski, Andrey Korshunov, Marco Stein, Thidathip Wongsurawat, Pornsuk Cheunsuacchon, Sith Sathornsumetee, Christian Koelsche, Clinton Turner, Emilie Le Rhun, Angelika Mühlebner, Philippe Schucht, Koray Özduman, Takahiro Ono, Hiroaki Shimizu, Marco Prinz, Till Acker, Christel Herold-Mende, Tobias Kessler, Wolfgang Wick, David Capper, Pieter Wesseling, Felix Sahm, Andreas von Deimling, Christian Hartmann, David E. Reuss

**Affiliations:** 1 Department of Neuropathology, Institute of Pathology, Heidelberg University Hospital, Heidelberg, Germany; 2 Clinical Cooperation Unit Neuropathology, German Cancer Research Center (DKFZ), German Consortium for Translational Cancer Research (DKTK), Heidelberg, Germany; 3 Department of Neurological Surgery, Helen Diller Research Center, University of California San Francisco, San Francisco, CA USA; 4 Institute of Pathology, Lausanne University Hospital and University of Lausanne, Lausanne, Switzerland; 5 Department of Neuropathology, Charité–Universitätsmedizin Berlin, Corporate Member of Freie Universität Berlin and Humboldt-Universität zu Berlin, Berlin, Germany; 6 German Cancer Consortium (DKTK), Partner Site Berlin, German Cancer Research Center (DKFZ), Heidelberg, Germany; 7 Institute of Neuropathology, University of Giessen, Giessen, Germany; 8 Department of Cellular Pathology, Queen Elizabeth Hospital/University Hospitals Birmingham, Birmingham, UK; 9 Institute of Neuropathology, Faculty of Medicine, University of Freiburg, Freiburg, Germany; 10 Department of Neurosurgery, University Hospital Gießen, Giessen, Germany; 11 Faculty of Medicine Siriraj Hospital, Mahidol University, Bangkok, Thailand; 12 Institute of Pathology, University Hospital Heidelberg, Heidelberg, Germany; 13 Centre for Brain Research, Department of Anatomy and Medical Imaging, Faculty of Medical and Health Sciences, University of Auckland, Auckland, 1023 New Zealand; 14 Department of Anatomical Pathology, LabPlus, Auckland City Hospital, Auckland, 1023 New Zealand; 15 Department of Neurology and Brain Tumor Center, University Hospital, University of Zurich, Frauenklinikstrasse 26, 8091 Zurich, Switzerland; 16 Department of Neurosurgery, University Hospital, University of Zurich, Frauenklinikstrasse 10, 8091 Zurich, Switzerland; 17 Department of Neuro Pathology, Amsterdam UMC, Location AMC, University of Amsterdam, Meibergdreef 9, 1105 Amsterdam, The Netherlands; 18 Department of Neurosurgery, Inselspital, Bern University Hospital, University of Bern, 3010 Bern, Switzerland; 19 Department of Neurosurgery, Acıbadem University, School of Medicine, Istanbul, Turkey; 20 Department of Neurosurgery, Akita University Graduate School of Medicine, Akita, Japan; 21 Centre for NeuroModulation (NeuroModBasics), University of Freiburg, Freiburg, Germany; 22 Signalling Research Centres BIOSS and CIBSS, University of Freiburg, Freiburg, Germany; 23 Department of Neurosurgery, University Hospital Heidelberg, Heidelberg, Germany; 24 Clinical Cooperation Unit Neurooncology, German Consortium for Translational Cancer Research (DKTK), German Cancer Research Center (DKFZ), Heidelberg, Germany; 25 Department of Neurology and Neurooncology Program, National Center for Tumor Diseases, Heidelberg University Hospital, Heidelberg, Germany; 26 Laboratory for Childhood Cancer Pathology, Princess Máxima Center for Pediatric Oncology, Utrecht, The Netherlands; 27 Department of Pathology, Amsterdam University Medical Centers/VUmc and Brain Tumor Center Amsterdam, Amsterdam, The Netherlands; 28 Hopp Children’s Cancer Center (KiTZ), Heidelberg, Germany; 29 Department of Neuropathology, Institute of Pathology, Hannover Medical School (MHH), Carl-Neuberg-Str. 1, 30625 Hannover, Germany

**Keywords:** Oligosarcoma, Oligodendroglioma, Gliosarcoma, 1p/19q, Codeletion, SMA, YAP1, NF1, TP53, TERT, DNA methylation, Type, Subtype, Variant, Prognosis

## Abstract

Oligodendrogliomas are defined at the molecular level by the presence of an IDH mutation and codeletion of chromosomal arms 1p and 19q. In the past, case reports and small studies described gliomas with sarcomatous features arising from oligodendrogliomas, so called oligosarcomas. Here, we report a series of 24 IDH-mutant oligosarcomas from 23 patients forming a distinct methylation class. The tumors were recurrences from prior oligodendrogliomas or developed de novo. Precursor tumors of 12 oligosarcomas were histologically and molecularly indistinguishable from conventional oligodendrogliomas. Oligosarcoma tumor cells were embedded in a dense network of reticulin fibers, frequently showing p53 accumulation, positivity for SMA and CALD1, loss of OLIG2 and gain of H3K27 trimethylation (H3K27me3) as compared to primary lesions. In 5 oligosarcomas no 1p/19q codeletion was detectable, although it was present in the primary lesions. Copy number neutral LOH was determined as underlying mechanism. Oligosarcomas harbored an increased chromosomal copy number variation load with frequent *CDKN2A/B* deletions. Proteomic profiling demonstrated oligosarcomas to be highly distinct from conventional CNS WHO grade 3 oligodendrogliomas with consistent evidence for a smooth muscle differentiation. Expression of several tumor suppressors was reduced with NF1 being lost frequently. In contrast, oncogenic YAP1 was aberrantly overexpressed in oligosarcomas. Panel sequencing revealed mutations in *NF1* and *TP53* along with *IDH1/2* and *TERT* promoter mutations. Survival of patients was significantly poorer for oligosarcomas as first recurrence than for grade 3 oligodendrogliomas as first recurrence. These results establish oligosarcomas as a distinct group of IDH-mutant gliomas differing from conventional oligodendrogliomas on the histologic, epigenetic, proteomic, molecular and clinical level. The diagnosis can be based on the combined presence of (a) sarcomatous histology, (b) IDH-mutation and (c) *TERT* promoter mutation and/or 1p/19q codeletion, or, in unresolved cases, on its characteristic DNA methylation profile.

**Supplementary Information:**

The online version contains supplementary material available at 10.1007/s00401-021-02395-z.

## Introduction

Oligodendroglioma is defined as a diffusely infiltrating glioma with an IDH mutation and codeletion of chromosomal arms 1p and 19q [23]. The upcoming WHO classification will recognize the following two malignancy grades: CNS WHO grade 2 and grade 3, no longer recommending the terminology of “anaplastic oligodendroglioma” for high-grade tumors. Grade 3 tumors are histologically defined by brisk mitotic activity and/or microvascular proliferation. Additionally, in oligodendrogliomas of histopathological unclear grade, it is recommended to evaluate the *CDKN2A/B* status and to use homozygous deletion as reason to assign grade 3 [23]. In contrast to IDH-mutant astrocytomas, oligodendrogliomas typically show retained expression of ATRX and no p53 accumulation, corresponding to an absence of mutations in these genes [33, 36]. Furthermore, oligodendrogliomas exhibit a typical DNA methylation profile with a high degree of global DNA methylation [11]. Recently, oligodendrogliomas were shown to display a unique histone-modification pattern with a reduced trimethylation of lysin 27 of histone H3 (H3K27me3) in comparison to IDH-mutant astrocytomas [17, 18, 28]. Oligodendrogliomas are associated with the most favorable prognosis among all diffuse gliomas in adults with a median overall survival of more than 14 years for patients receiving combined radio-chemotherapy [44].

Sarcomatous features are rare in glial tumors and most commonly encountered in gliosarcomas, which is nowadays considered as a variant of glioblastoma, IDH-wildtype [23]. However, sarcomatous differentiation may rarely occur in other glial neoplasm as well but has not been associated with specific molecular or clinical features. Rubinstein first described an oligodendroglioma with foci of a sarcoma in 1972 [35]. Several case reports from the morphologic era confirmed such so-called “oligodendroglioma-sarcomas” that developed from oligodendrogliomas. It was speculated that the sarcoma component of these tumors were malignant transformed endothelia or perivascular fibroblasts induced secondary to oligodendroglioma [1, 16, 27]. Later case reports also included information on the 1p/19q and sometimes even the IDH status of such tumors, which were then mostly referred to as “oligosarcomas” [19, 34, 38, 41, 42]. The WHO classification describes sarcomatous areas as a rare pattern in grade 3 oligodendrogliomas [23]. The largest cohort to date was collected by Rodriguez et al., consisting of 7 cases harboring oligodendroglial and sarcomatous components with smooth muscle actin (SMA) expression. As the study was conducted in 2007, IDH status was not evaluated and 1p/19q codeletion was only detected in 5 cases in the oligodendroglial compartment [34]. Such loss of 1p/19q codeletion in the secondary sarcomatous component with persisting detectable IDH mutation compared to the primary oligodendroglioma was confirmed in one case report [19]. In contrast, three other case reports also documented a combined deletion of 1p/19q as well in the secondary sarcomatous component with a stable IDH mutation status [38, 41, 42].

Here, we report a series of 24 oligosarcomas from 23 patients with for 12 cases matched primary oligodendroglioma. We demonstrate that oligosarcomas are highly distinct and follow an aggressive clinical course.

## Material and methods

### Tissue samples

Tissue samples were collected from university hospitals in Akita, Amsterdam, Auckland, Bangkok, Berlin, Bern, Birmingham, Freiburg, Gießen, Hannover, Heidelberg, Hongkong, Istanbul, Lille, Moscow and Utrecht. Tissue and data collection were performed in consideration of local ethics regulations and approval. Case 17 derived from TCGA; therefore, the results shown here are in part based upon data generated by the TCGA Research Network: https://www.cancer.gov/tcga.

### DNA extraction

Tumor DNA was extracted from areas with highest tumor cell content using the automated Maxwell system with the Maxwell 16 Tissue DNA Purification Kit or the Maxwell 16 FFPE Plus LEV DNA Purification Kit (Promega, Madison, USA), according to the manufacturer’s instructions. DNA-extraction from EDTA blood was done using the Maxwell RSC Blood DNA Kit (Promega). DNA concentrations were determined using the Invitrogen Qubit dsDNA BR Assay Kit (Thermo Fisher Scientific, Waltham, USA) on a FLUOstar Omega Microplate Reader (BMG Labtech GmbH, Ortenberg, Germany).

### DNA methylation and t-SNE analysis

DNA methylation profiles were generated using the Infinium HumanMethylation450 (450 k) BeadChip or Infinium MethylationEPIC (850 k) BeadChip array (Illumina, San Diego, USA) according to the manufacturer’s instructions. The data were processed as previously described [11]. The t-SNE plot was computed via the R package Rtsne using the 25,000 most variable CpG sites according to standard deviation, 3000 iterations and a perplexity value of 10. We chose samples from Heidelberg with highest scores in the brain tumor classifier and from previously published series as reference cases.

### Data availability

**Fig. 1:**
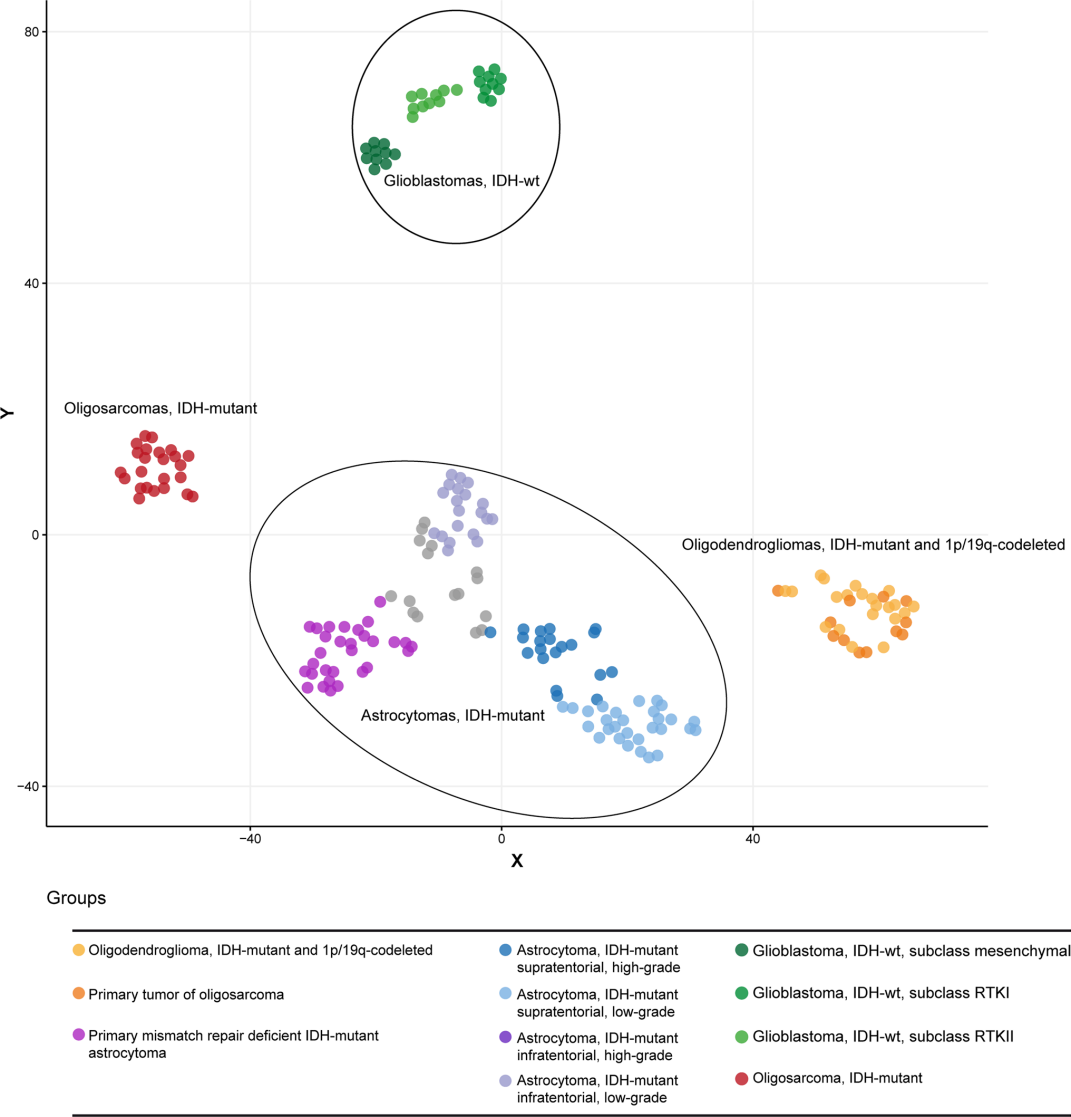
Oligosarcomas are epigenetically distinct. Unsupervised DNA methylation-based t-SNE for 24 oligosarcomas, 12 primary tumors of oligosarcomas and 166 reference cases. Each dot represents an individual tumor sample. RTK = receptor tyrosine kinase; wt = wildtype

All raw and processed IDAT files from Fig. 1 as well as the SNP array data are available via the Gene Expression Omnibus (GEO) database with the accession number GSE190365. The proteomic data are available via the ProteomeXchange consortium with the accession number PXD030442.

## SNP array

Zygosity-sensitive copy number analysis were generated using the HumanCytoSNP-12 BeadChip V2.1(Illumina, San Diego, USA) according to the manufacturer’s instructions. Analyses of raw data and visualisation was done as described by Popova et al. [29]. SNP array data were processed using the Genotyping module in the GenomeStudio Software Version v2.0.

### MGMT promoter methylation analysis

MGMT promoter methylation status was calculated from the 450 k/850 k data as described by Bady et al. [5].

### Copy number profiling

Copy number profiles were generated as previously described [39]. The *CDKN2A/B* locus was evaluated manually as chromosomal deletions were often too small to be identified and plotted automatically in the summarized copy number plot.

### Sample preparation of FFPE tissue for mass spectrometry

Punches of solid tumor tissues were mechanically disintegrated in a bead tube (Bertin Technologies SAS, Montigny Le Bretonneux, France) filled with ethanol for bead milling using a Precellys 24 (Bertin Technologies SAS, Montigny Le Bretonneux, France). Deparaffination was done using xylene and ethanol. Lysis was carried out under pressure cycling using a Barocycler 2320EXT (60 cycles, 45,000 psi for 50 s per cycle and 14.7 psi for 10 s per cycle, 95 °C). After protein quantification with BCA-Assay (Pierce BCA Protein Assay Kit, Thermo Scientific, Waltham, MA) samples were reduced and alkylated by adding Tris (2-carboxyethyl) phosphine (10 mM) and iodoacetamide (40 mM) (30 min in the dark at 25 °C). Proteins were precipitated by adding ice-cold acetone (80%) to the solution and incubating at − 20 °C for 1 h. Trypsin/Lys-C Mix (Promega, Madison, WI) was used for digestion with an enzyme-to-substrate-ratio of 1:25. Digestion was carried out under pressure cycling in a Barocycler 2320EXT (Pressure Biosciences, Easton, MA) (150 cycles, 45,000 psi for 50 s per cycle and 14.7 psi for 10 s per cycle at 33 °C). Samples were acidified with 1% trifluoroacetic acid and dried in a Concentrator Plus Speed Vac (Eppendorf, Hamburg, Germany) until dry at 30 °C. Dried pellets were dissolved in a solution of 0.1% trifluoracetic and 2.5% hexafluoroisopropanol using an ultrasonic bath. The peptide solution was quantified using Pierce Quantitative Colorimetric Peptide Assay (Thermo Scientific Waltham, MA).

### Liquid chromatography and LC–MS/MS acquisition

LC–MS samples were separated using an UltiMate 3000 HPLC. Prior separation samples were directed through an Acclaim PepMap 100 trap column (C18 100 Å, 5 µm, 300 µm × 55 mm). Peptides were separated on nanoLC nanoEase 200 mm M/Z BEH C18, 130 Å, 1.7 µm column. For each analysis we loaded 1 µg of peptides onto the column and separated them using a 3.5 h gradient. This was set as follows: at 2% of B for 3 min, from 2 to 8% B in 15 min, from 8 to 25% B in 125 min, from 25 to 40% B in 30 min, from 40 to 95% B in 1 min, 5 min at 95% B, from 95 to 2% B in 1 min, 30 min at 2%B. Solvent A contained 99.9% H2O and 0.1% FA, solvent B contained 80% ACN, 19,9% H2O and 0,1% FA. The HPLC was coupled online to an Orbitrap Exploris™ 480 Mass Spectrometer. The Sample was ionized using 2.2 kV spray voltage and a capillary temperature of 275 °C. The Mass spectrometer was operated in data-dependent mode to automatically measure MS1 and MS2. Full scan MS1 Spectra were acquired at a resolution of 120.000 at 400 m/z in the orbitrap covering the mass range of 375–1700 m/z. MS2 spectra were acquired at a resolution of 30.000 at 400 m/z in cycles after every full scan, where the 20 most abundant precursor ions with a normalized collision energy of 27 were selected for fragmentation.

### Peptide identification

The raw data were analysed in the MaxQuant environment with default settings (version 1.6.17.0) [14]. The data was searched against the human Uniprot database (August 2018 release). Label-free quantification was performed with the MaxLFQ algorithm and a minimum ratio count of one. If proteins could not be distinguished on the basis of unique peptides, they were merged by MaxQuant as one protein group.

### Data analyses

Post-processing and statistical analysis were carried out by the statistical programming language R (version 4.0.4). Proteins with more than 10% missing values within a condition were discarded to reduce data missingness which could interfere with upcoming statistical analysis. The functions from the R-package ‘DEP’ were used for normalization, missing value imputation and for differential expression analysis. Here, technical variability between samples was corrected with vsn. After normalization, missing values substituted by using a downshift imputation (shift = 1.5, scale = 0.5). Differential expression was examined by the R-package ‘limma’ and Benjamini-Hochberg (BH) was used for false discovery rate (FDR) of the resulting p-values. Enrichment examination was performed with the R-package ‘pathfindR’ with proteins obtained from differential expression in pathways via active protein interaction networks (PIN). Proteins with adjusted *p* values < 0.01 were used for active subnetwork search against PINs from the STRING database and gene sets from the KEGG database were used for the later enrichment analysis.

### Gene sequencing and mutational burden

For 18 cases (including primary lesions) next-generation sequencing was performed on a NextSeq sequencer 500 (Illumina) as described previously [37]. Libraries were enriched by hybrid capture with custom biotinylated RNA oligo pools covering exonic regions 171 genes known for frequent alterations in brain tumors.

### Immunohistochemistry

Immunohistochemical analyses was conducted as previously described [39]. Primary antibodies were diluted as follows: IDH1 R132H (1:2, clone H09 [12]), NF1 (1:4, clone NFC [32]), p53 (1:50, Novocastra, Leica, Wetzlar, Germany), SMA (1:500, Cell Marque, Sigma Aldrich, St. Louis, USA), H3K27me3 (1:25, monoclonal antibody, Cell signalling, Danvers, USA), YAP1 (1:100, Cell signalling), Caldesmon (CALD1) (1:100, clone E89, zytomed-systems, Berlin, Germany), GFAP (1:2000, clone GA5, Cell signalling, Danvers, USA), OLIG2 (1:50, clone ERP 2673, Abcam, Cambridge, UK). Stained slides were scanned on the Aperio AT2 Scanner (Aperio Technologies, Vista, USA) and digitalized using Aperio ImageScope software v12.3.2.8013.

### Statistical analysis

Differences in *CDKN2A* deletion frequencies were tested for significance with chi-square and CNV load between groups were tested for significance with unpaired *t*-tests. Kaplan–Meier curves were created and log-rank tests calculated using GraphPad Prism 9 for Windows, GraphPad Software, San Diego, California USA, https://www.graphpad.com. A *p* value ≤ 0.05 was considered as significant. The Pearson correlation was calculated on the B-allele Frequencies (BAF) to estimate the genetic relationship between tumors coming from the same and different patients.

## Results

### Oligosarcomas form a distinct DNA methylation group

We initially performed DNA methylation analysis on nine pairs of primary oligodendroglioma and the recurrent tumor with sarcomatous features. Results for the primary oligodendrogliomas from the brain tumor classifier showed matching calibrated scores (> 0.9) for “Oligodendroglioma, IDH-mutant and 1p/19q-codeleted”. In contrast, most of the recurrent tumors with sarcomatous features showed elevated scores for “IDH glioma, subclass high-grade astrocytoma“ and some samples reached a matching score (> 0.9) for this reference set. However, comparing these methylation profiles with an extensive cohort of more than 75,000 tumors from various entities, we found the profile of sarcomatous tumors to be highly distinctive while the profiles of the primary oligodendrogliomas fell together with conventional oligodendrogliomas (data not shown). By this comparison we identified 15 additional samples from 14 patients which had the same methylation profile as the sarcomatous tumors. In restricted analyses using t-distributed stochastic neighbor embedding (t-SNE)-analysis we confirmed the distinctiveness of the DNA methylation profile of the sarcomatous tumors which were clearly separated from their primary tumor manifestations and from conventional oligodendrogliomas, as well as from IDH-wildtype glioblastomas, from high- and low-grade supra- and infratentorial IDH-mutant astrocytomas, and from primary mismatch repair deficient IDH-mutant astrocytomas (PMMRDIA) (Fig. 1, Supplementary tables, online resource). Like Rodriguez et al. [34], we called this novel methylation group “oligosarcomas”. Mean DNA methylation levels at differentially methylated positions were significantly reduced in oligosarcomas compared to oligodendrogliomas (Supplementary Fig. 1, online resource), while the *MGMT* promoter was methylated in all cases.

### Oligosarcomas show extensive presence of reticulin fibers, accumulation of p53, re-gain of H3K27me3 and loss of OLIG2

**Fig. 2:**
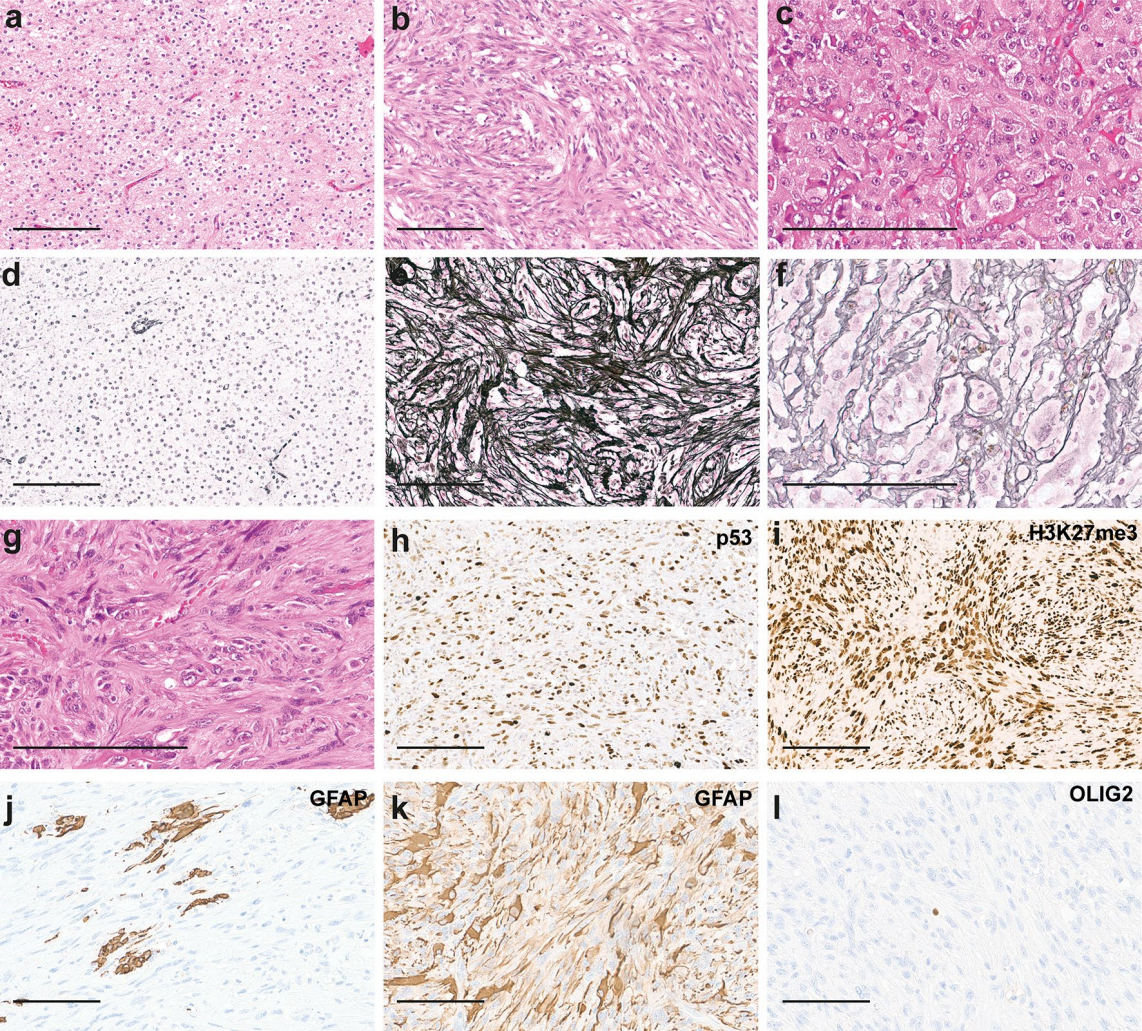
Histology and immunohistochemistry of oligosarcomas. HE staining of the primary tumor (**a**) and oligosarcoma (**b**) of patient 3. The oligosarcoma of patient 16 shows highly anaplastic tumor cells in HE staining (**c**). Reticular fibers are restricted to blood vessels in Gomori’s reticulin staining in the oligodendroglioma of patient 3 (**d**) but extensively present between tumor cells in the oligosarcoma (**e**). Groups of tumor cells of the tumor shown in c are also embedded in reticulin fibers (**f**). Patient 8 presented with an oligosarcoma (g; HE), the tumor cell nuclei showing high p53 (**h**) and retained H3K27me3 expression (**i**). Oligosarcoma with subtotal loss (**j**) or mostly retained (**k**) expression of GFAP. Lack of OLIG2 expression in oligosarcoma with single entrapped positive nuclei (**l**). Scale bar is 200 µm

Histologically, most samples were diagnosed as oligosarcoma (9/24, 38%) or anaplastic oliogodendroglioma (8/24, 33%). Two cases were initially diagnosed as gliosarcoma, two cases as anaplastic astrocytoma, one case as anaplastic astrocytoma with sarcomatous component, one case as glioblastoma and one sample as leiomyosarcoma (Table 1, Supplementary tables, online resource). All samples analysed presented with sarcomatous features (Fig. 2). Using Gomori’s reticulin staining the embedding of single or small groups of tumor cells in a dense network of reticular fibers was visible in all cases (Fig. 2e, f). The tumor cells were either spindle shaped with often vacuolated nuclei or epithelioid with prominent nucleoli (Fig. 2b, c, g). Brisk mitotic activity and pseudopalisading necrosis were common. Cases with surrounding parenchyma showed a relatively sharp demarcation of the tumor rather than diffuse infiltrative growth. In one case (patient 3) areas of conventional oligodendroglioma without sarcomatous features were present which were clearly delineated from the sarcomatous regions. Proliferation measured by Ki67 staining varied from 10 to 80% with a mean of 40.9%. All but one oligosarcomas were positive for IDH1-R132H. ATRX was retained in all cases analyzed. All but one sample (11/12, 91.7%) showed extensive accumulation of p53 which was not present in the primary oligodendrogliomas (Fig. 2h). Interestingly, nuclear H3K27me3 expression, which is usually lost in oligodendrogliomas as it was in the primary oligodendroglioma samples (4/4), was abundantly present in all but one oligosarcoma (10/11, 90.9%; detected by monoclonal antibody clone C36B11, Fig. 2i). GFAP and OLIG2 were positive in all primary tumors (10/10) with one tumor showing a mixture of positive and negative cells whereas all others showed widespread positivity. Oligosarcomas were negative for OLIG2 except one case showing an intermingled mixture of positive and negative cells (13/14). GFAP was variable expressed in oligosarcomas. While some oligosarcomas retained positivity in many tumor cells, others were mostly negative and yet others displayed a patchy GFAP expression pattern (Fig. 2j, k).

**Table 1:** Clinico- pathological data of oligosarcomas

Patient	Age	Sex	Localization	1p/19q	IDH	Histological diagnosis	P/R	WHO grade P	OS [days]	OS since first diagnosis [days]	Death
1	48	m		co-del	IDH1 R132H	Gliosarcoma WHO grade IV	R	II/III	902	7472	Yes
2	50	m		co-del	IDH1 R132H	Oligosarcoma	R	II	2441	2855	No
3	39	m	frontal	co-del	IDH1 R132H	Oligosarcoma	R	II	1051	2876	No
4	55	m		co-del	IDH1 R132H	Oligosarcoma	R	II		1825	No
5	40	m	frontal	co-del	IDH1 R132H	Oligosarcoma	R	III	1904	3551	Yes
6	56	f	temporal	co-del	IDH1 R132H	Oligosarcoma	R	III/III	274	4652	Yes
7	68	m	frontal	co-del	IDH1 R132H	Glioblastoma WHO grade IV	P		1366	1366	Yes
8	59	m	frontal	co-del	IDH2 R172W	Oligosarcoma	R	II	408	8893	Yes
9	24	f	temporal	co-del	IDH1 R132H	Anaplastic oligodendroglioma WHO grade III	R	III	180	2342	Yes
10	48	f	frontal	co-del	IDH1 R132H	Anaplastic oligodendroglioma WHO grade III	R	IV	420	420	No
11	51	f	frontal	wt	IDH1 R132H	Gliosarcoma WHO grade IV	R	II	3680	6600	No
12	46	m	frontal	co-del	IDH1 R132H	Leiomyosarcoma	R	III		2376	No
13	57	m		wt	IDH1 R132H	Oligosarcoma	R	II		1095	No
14	61	m	temporal	co-del	IDH1 R132H	Anaplastic oligodendroglioma WHO grade III	R	II/II		3103	No
15	46	m	parietal	co-del	IDH1 R132H	Anaplastic oligodendroglioma WHO grade III	P		1200	1200	Yes
15	48	m	parietal	co-del	IDH1 R132H	Anaplastic oligodendroglioma WHO grade III	R	III			
16	58	f	hemispheric	co-del	IDH1 R132H	Anaplastic oligodendroglioma WHO grade III	R	II		8787	No
17	38	f		co-del	IDH1 R132H	Anaplastic astrocytoma WHO grade III	P				
18	79	f	parietal	co-del	IDH1 R132H	Oligosarcoma	R	unknown	365	11,680	No
19	50	m		co-del	IDH1 R132H	Oligosarcoma	R	II	178	3446	Yes
20	42	m	frontotemporal	wt	IDH1 R132H	Anaplastic oligodendroglioma WHO grade III	R	II	128	4268	Yes
21	46	f	temporal	19q del	IDH1 R132H	Anaplastic oligodendroglioma WHO grade III	R	II	559	6570	Yes
22	60	m		co-del	IDH1 R132H	Anaplastic astrocytoma WHO grade III	P		972	972	Yes
23	58	m	temoroparietal	19q del	IDH1 R132H	Anaplastic astrocytoma WHO grade III with sarcomatoid component	R	III	670	4047	Yes

*P*  primary tumor; *R* recurrent tumor

### Oligosarcomas may lose 1p/19q codeletion but retain copy number neutral 1p/19q loss of heterozygosity

**Fig. 3:**
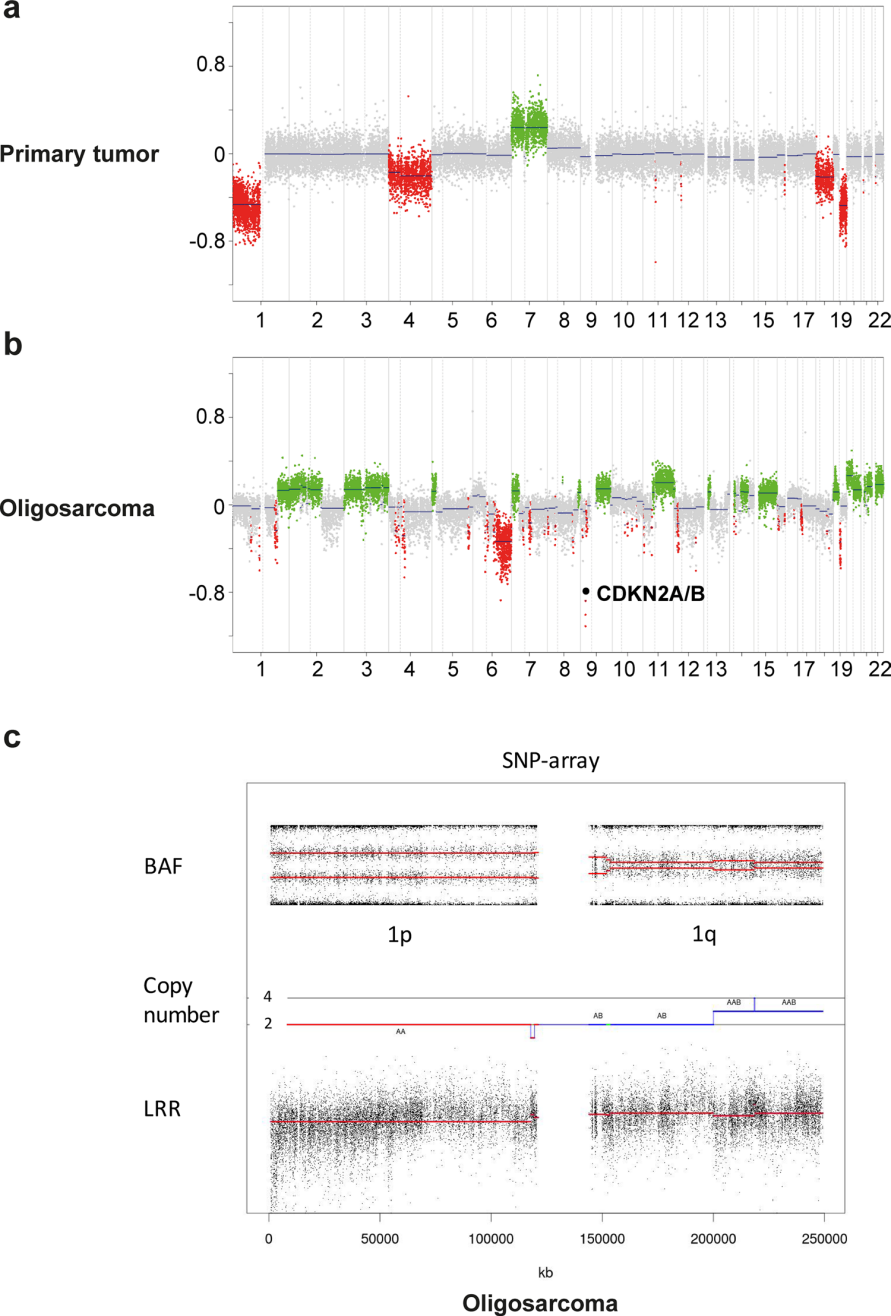
Oligosarcomas may lose 1p/19q codeletion but retain CN-LOH. Copy number profiles of the tumors of patient 11: The primary tumor shows a complete 1p/19q codeletion (**a**) while in the subsequent oligosarcoma 1p deletion is absent but homozygous *CDKN2A/B* loss is present (**b**). SNP-array data regarding chromosome 1 shows deviation of BAF in 1p while the log R ratio (LRR) shows presence of two copies of the arm demonstrating CN-LOH. Note also the partial trisomy of 1q

Next, we assessed the 1p/19q status of the tumors. All primary lesions showed complete 1p/19q codeletion as conventional oligodendrogliomas do. However, four oligosarcomas lost the 1p/19q codeletion that was present in the primary tumor (Fig. 3a, b). A fifth recurrent oligosarcoma displayed an incomplete 1p/19q codeletion but there was not enough material left from the primary tumor for a direct comparison. To further dissect the chromosomal state of oligosarcomas we used single nucleotide polymorphism (SNP)-array analyses of six patient-matched pairs of primary oligodendroglioma and recurrent oligosarcoma including three samples without complete 1p/19q codeletion. Results confirmed 1p/19q codeletion in all primary oligodendrogliomas and in 3 oligosarcomas. Most importantly, all three oligosarcomas without 1p deletion displayed allelic imbalance in the B-allele frequency (BAF) without or incomplete deletion in the log-R ratio, demonstrating copy number neutral (CN) loss of heterozygosity (LOH) (Fig. 3c). A CN-LOH was also present for 19q in one of the oligosarcomas without 19q deletion. Further analyses revealed that the BAF for SNPs on 1p are highly correlated between primary oligodendroglioma and recurrent oligosarcoma consistent with a clonal relation (Supplementary Fig. 2, online resource). Of note, 3/6 oligosarcoma displayed a tetraploid genome whereas all primary oligodendrogliomas were diploid (all SNP plots are given in Supplementary Figs. 3, 4, 5, online resource). Interestingly, 5/6 oligosarcomas showed more than 1 copy of chromosome 1p which is the typical constellation in oligodendroglioma. Oligosarcomas presented either with a relative 1p deletion with 3 copies on a tetraploid background or with two copies and copy number neutral LOH.

### Oligosarcomas frequently harbor 6q loss and *CDKN2A/B* deletion

**Fig. 4:**
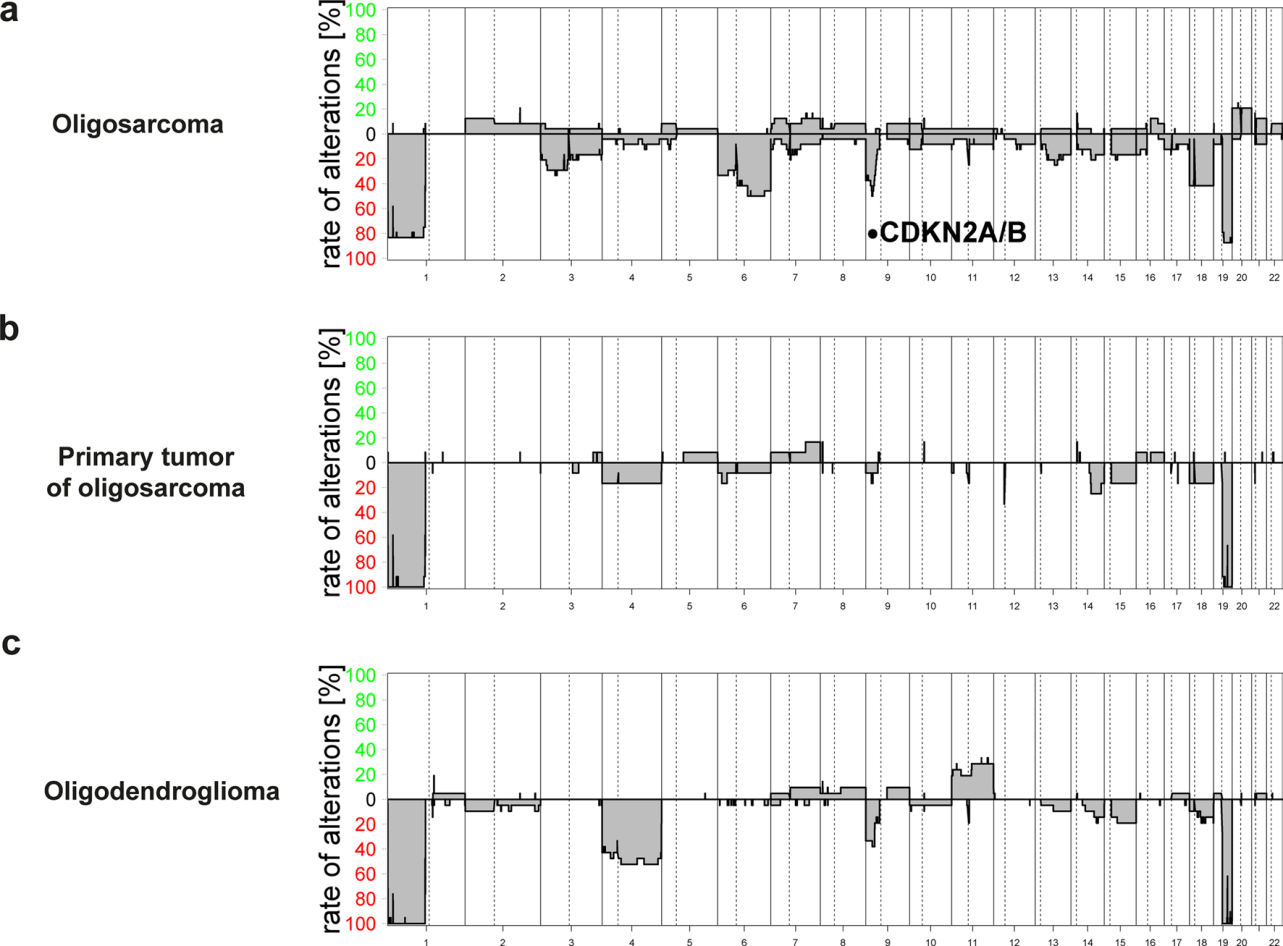
Oligosarcomas have distinct copy number profiles. Summary of chromosomal copy number plots of oligosarcomas (**a**, *n* = 24), primary tumor of oligosarcoma (**b**, *n* = 12) and conventional oligodendroglioma (**c**, *n* = 21). In oligosarcomas, homozygous *CDKN2A/B* deletion and loss of chromosome 6q are frequent, and in a few cases 1p/19q loss is not observed (**a**). Copy number profiles of primary tumors of oligosarcomas and conventional oligodendrogliomas are similar (**b**, **c**)

Assessing general chromosomal copy number alterations, we noted that oligosarcomas presented with a high frequency of chromosome 6q loss (50%) which was not detected in oligodendrogliomas and just in one primary tumor subclonally (Fig. 4). Monosomies of chromosome 3 and 18 were present in 30% and 40%, respectively, of oligosarcomas but were absent or rare in oligodendrogliomas. There was a significant increase in *CDKN2A/B* locus deletions on 9p compared to primary lesions (20/24, 83% compared to 3/12, 25%, *p* = 0.000593, chi-square). Deletions of *CDKN2A/B* were mostly homozygous (15/24, 63%). Of note, in two cases of oligosarcomas presenting with heterozygous deletion of *CDKN2A/B* this was not present in the primary oligodendroglioma. Overall, compared to oligodendrogliomas oligosarcomas had a higher frequency of copy number alterations (*p* = 0.029, unpaired t-test), and in particular showed an increase in chromosomal losses. There was a paucity of amplifications and a *YAP1-* amplification in the tumor of patient 23 was the only oncogene-amplification detected in the cohort (Supplementary Fig. 6, online resource).

### The proteome of oligosarcomas is highly distinct demonstrating smooth muscle differentiation

**Fig. 5:**
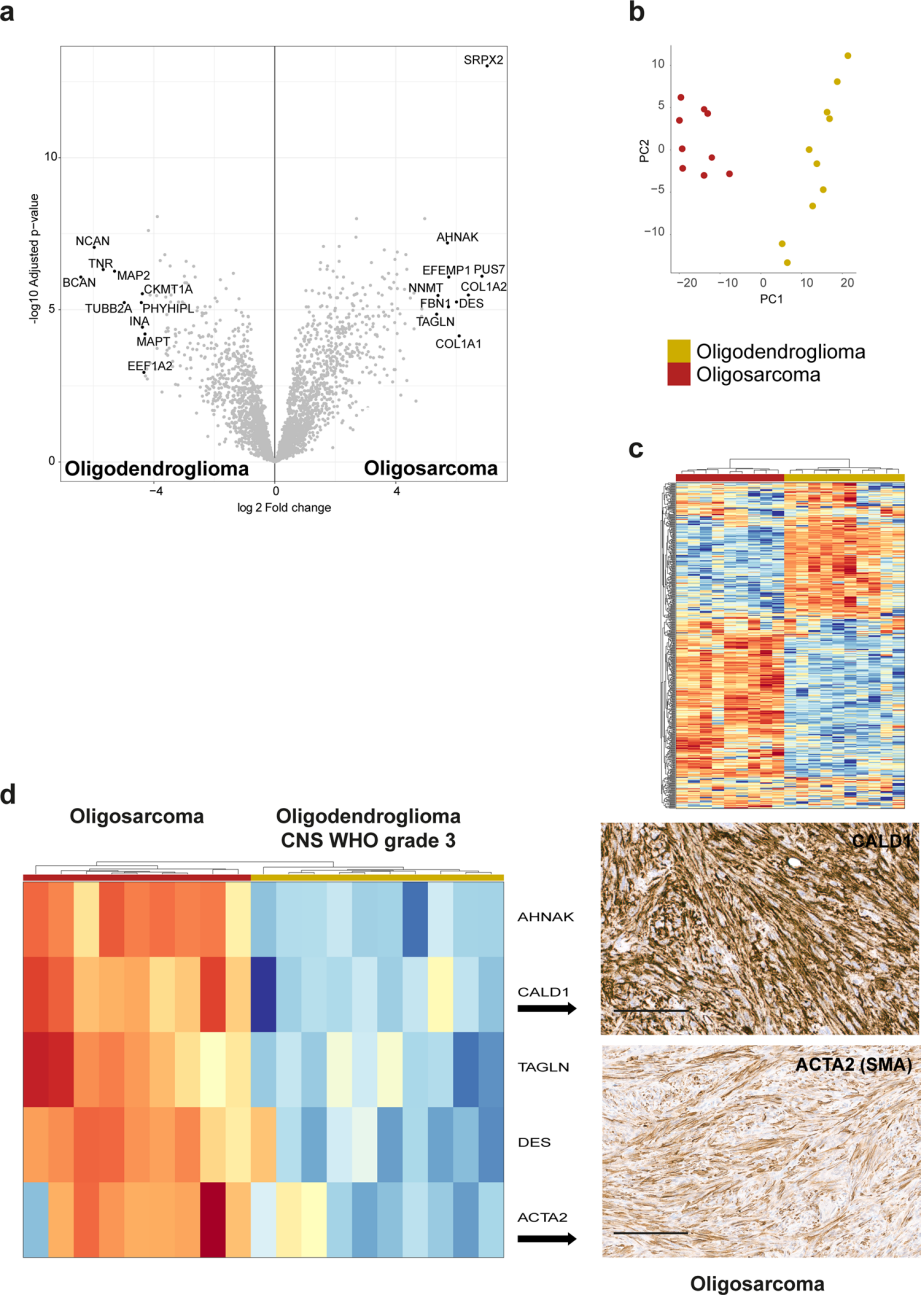
Oligosarcomas have a highly distinct proteome with evidence for smooth muscle differentiation. **a** Volcano plot of significantly differentially regulated proteins between oligosaroma and conventional grade 3 oligodendroglioma. Using the 500 most variable proteins of the dataset for principal component analysis (**b**) and unsupervised hierarchical clustering (**c**) differentiates oligosarcomas from conventional grade 3 oligodendrogliomas. **d** Heatmap shows upregulation of (smooth) muscle specific proteins in oligosarcomas (left) and immunohistochemistry confirms expression of CALD1 and ACTA2 (SMA) in tumor cells of oligosarcoma (right). Scale bar is 200 µm

According to the WHO classification oligosarcomas are a histological pattern of grade 3 oligodendrogliomas [23]. In order to get a deeper insight into their relationship we performed deep proteomic profiling of 9 oligosarcomas and 10 conventional CNS WHO grade 3 oligodendrogliomas using liquid chromatography coupled mass spectrometry (LC/MS). Differential protein abundance analyses identified 896 significantly differentially abundant proteins, with 540 being upregulated and 356 being downregulated in oligosarcoma (Fig. 5a, Supplementary table 1, online resource). Principal component analyses and unsupervised hierarchical clustering clearly separated the proteomes of oligosarcoma and grade 3 oligodendrogliomas (Fig. 5b, c). Three of the top 10 upregulated proteins (TAGLN, AHNAK, DES) in oligosarcoma were muscle or smooth muscle specific proteins. ACTA2 (commonly referred to as smooth muscle actin (SMA)) was upregulated in all but one oligosarcoma. Furthermore, the smooth muscle marker protein caldesmon 1 (CALD1) was consistently upregulated in all oligosarcomas as well. Given that SMA and CALD1 are widely used in diagnostics, we performed immunohistochemistry with these markers, even though other proteins were even more strongly differentially abundant. Corresponding with LC/MS results, immunohistochemistry for SMA was indeed positive in all but one sample (7/8, 87.5%) and CALD1 was at least focally positive in all (10/10) (Fig. 5d). Taken together, these data suggested that at least a partial smooth muscle differentiation is common in oligosarcoma. Additional functional network analyses of the proteomic data revealed the strongest enrichment of proteins associated with the KEGG terms “ribosome”, “regulation of actin cytoskeleton”, “focal adhesion”, “protein processing in endoplasmic reticulum” and “shigelosis” suggesting altered activity of several major organelles in oligosarcoma (Supplementary Fig. 7, online resource).

### Oligosarcomas frequently lose NF1 and gain YAP1 expression

**Fig. 6:**
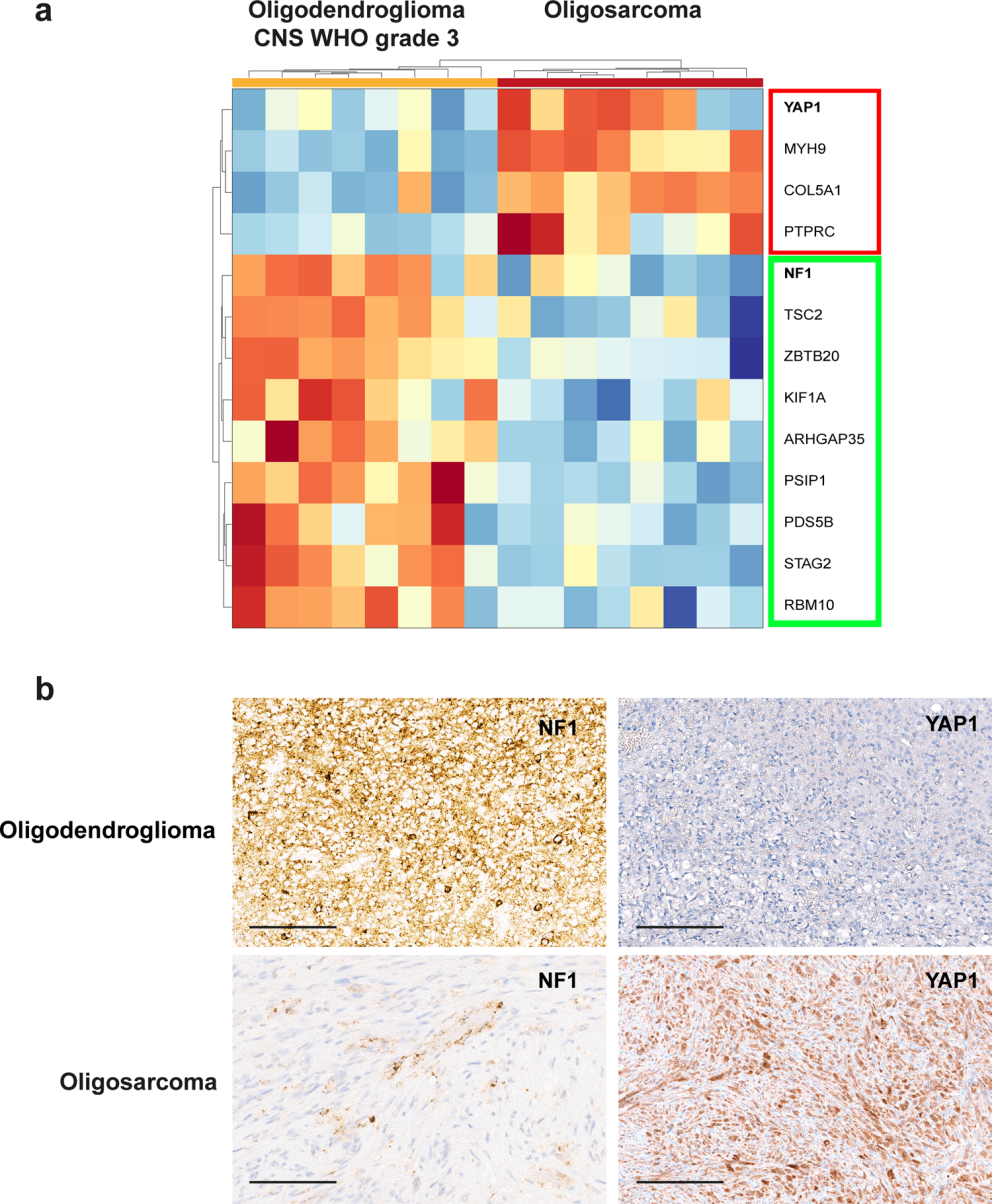
Oligosarcomas frequently lose NF1 and gain YAP1 expression. **a** Heatmap of selected cancer proteins in oligosarccomas shows downregulation of tumor suppressors (in green rectangle) and upregulation of oncoproteins (in red rectangle). **b** Immunohistochemistry shows retained expression of NF1 and lack of expression of YAP1 in an oligodendroglioma and loss of NF1 expression accompanied by strong YAP1 expression in an oligosarcoma. Scale bar is 200 µm

In an analysis focusing on known or suspected cancer drivers we identified 36 differentially abundant proteins. Considering downregulation only of relevance for tumor suppressive proteins and upregulation only for oncogenic proteins we identified 13 proteins of particular interest (Fig. 6a). NF1 and STAG2 were the most strongly downregulated candidates with well-established tumor suppressor functions. From four likely pro-tumorigenic proteins upregulated in oligosarcoma, YAP1 appeared as the most interesting candidate due to its well-established role as tumor driver in different types of cancer including sarcomas [20, 47]. In order to confirm these findings, we used immunohistochemistry to evaluate the protein prevalence in tumor cells. While immunohistochemistry for STAG2 did not provide evidence for a complete loss of expression in tumor cells, 7/11 (63.6%) oligosarcomas showed a tumor cell specific loss of NF1 expression (Fig. 5b). In contrast NF1 was found to be retained in primary lesions (9/9) and in 20/20 reference cases of conventional oligodendroglioma. YAP1 was strongly and widely positive in the cytoplasm and nucleus of tumor cells in 10/11 (91%) oligosarcomas (Fig. 6b), while only 1 out of 20 (5%) conventional oligodendrogliomas showed YAP1 staining, with only a subset of tumor cells being positive. Interestingly, in the case containing areas of conventional oligodendroglioma the YAP1 expression was strictly confined to the sarcomatous part of the tumor (Supplementary Fig. 8, online resource).

### Oligosarcomas harbor mutations in IDH, *TERT* promoter, *FUBP1*, *CIC*, *NF1* and *TP53*

We performed panel sequencing covering 171 genes frequently mutated in brain tumors for 13 oligosarcomas and 6 precursor tumors. Except for one oligosarcoma which harbored an *IDH2* (R172W) mutation, all other samples were *IDH1* (R132H) mutated. A hotspot *TERT* promoter mutation was detected in 11/13 samples, 5/13 samples had a *FUBP1* mutation, 4/13 samples a *CIC* mutation similar to conventional oligodendrogliomas. *NF1* mutations were detected in 2/13 samples. External reports stated an additional *NF1* stopgain mutation for the tumor of patient 18 that also presented with NF1 loss via immunohistochemistry. Detailed CNV analyses revealed deletions affecting the *NF1* locus in 6/24 oligosarcomas (25%), 3 of which were homozygous and 3 of which were heterozygous (Table 2, Supplementary tables, online resource). One oligosarcoma carried a hotspot *PIK3CA* (E542K) mutation whereas pathogenic *NOTCH1* mutations were not detected. Pathogenic mutations in *TP53* were present in two oligosarcomas. For patient 16, who was treated with alkylating agents before developing an oligosarcoma, the tumor was hypermutant with a mutational burden of 59 mutations per megabase. Beside two *MSH2* stopgain mutations, the tumor had two *TP53* stopgain mutations, three *NF1* missense variants and a *NF1* splicing variant while NF1 protein was lost. Using whole transcriptome sequencing of 4 oligosarcoma samples, no known or recurrent gene-fusion could be detected.

**Table 2:** Molecular data of oligosarcoma

Tumor	Patient	IDH	1p/19q	MGMT	TERT	CIC	FUBP	TP53	NF1	CDKN2A/B
1	1	R132H	Loss	meth	C228T	wt	wt	wt	wt, homo-del	het-del
2	2	R132H	Loss	meth	wt	wt	Stopgain	wt	wt, het-del	homo-del
3	3	R132H	Loss	meth	C250T	wt	wt	wt	wt, homo-del	homo-del
4	4	R132H	Loss	meth	C250T	wt	wt	wt	wt	homo-del
5	5	R132H	Loss	meth	C250T	fr del	wt	wt	wt, het-del	wt
6	6	R132H	Loss	meth	C228T	fr del	nonfr del	wt	wt	het-del
7	7	R132H	Loss	meth	C228T	wt	fr del	wt	splicing	homo-del
8	8	R172W	Loss	meth	C228T	wt	wt	wt	wt, homo-del	homo-del
9	9	R132H	Loss	meth	C228T	SNV	nonfr del	wt	wt, het-del	homo-del
10	10	R132H	Loss	meth						homo-del
11	11	R132H	wt	meth	C228T	wt	wt	SNV	wt	homo-del
12	12	R132H	Loss	meth	C228T	wt	wt	wt	wt	homo-del
13	13	R132H	Wt	meth						wt
14	14	R132H	Loss	meth						homo-del
15	15	R132H	Loss	meth						het
16	15	R132H	Loss	meth						homo-del
17	16	R132H	Loss	meth	wt	wt	wt	Stopgain	Splicing, SNV	wt
18	17	R132H	Loss	meth		wt	wt	wt	wt	het
19	18	R132H	Loss	meth						homo-del
20	19	R132H	Loss	meth						homo-del
21	20	R132H	wt	meth						homo-del
22	21	R132H	19q loss	meth						wt
23	22	R132H	loss	meth	C228T					het-del
24	23	R132H	19q loss	meth	C228T	SNV	Splicing	wt	wt	homo-del

*pTERT* TERT promoter; *codel* codeletion; *meth*  methylated; *wt* wildtype; *fr del* frameshift deletion; *nonfr del* non-frameshift deletion; *SNV* single nucleotide variant; *het-del* heterozygous deletion; *homo-del* homozygous deletion

### Oligosarcomas are frequently preceded by oligodendrogliomas and show an aggressive clinical course

**Fig. 7:**
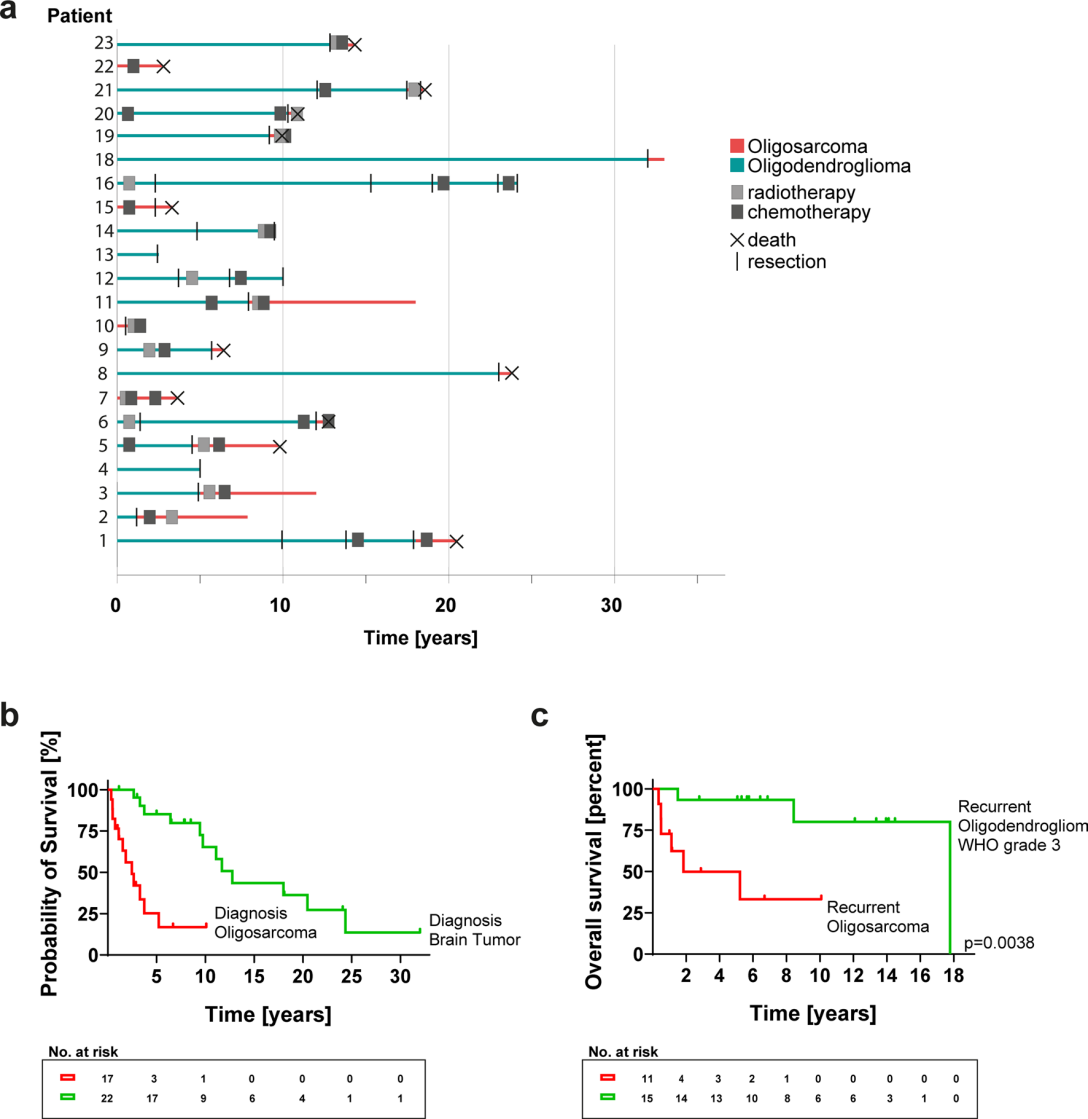
Clinical behavior of oligosarcomas. **a** Oligosarcomas can arise from oligodendrogliomas after different time spans and treatment procedures but may also occur de novo. **b** Kaplan–Meier analyses of the overall survival of patients with oligosarcoma, calculated from the timepoint of first diagnosis of a brain tumor (green) and from timepoint of oligosarcoma manifestation (red). **c** Kaplan–Meier analyses of the overall survival after a first progression event in form of a grade 3 oligodendroglioma (green) or an oligosarcoma (red)

Evaluation of the clinical data showed that the median age for patients diagnosed with an oligosarcoma was 50 years (range 24–79 years). There were 8 female and 15 male patients in our cohort (gender ratio 1:1.9) (Table 1). In addition to the 12 oligosarcomas for which the DNA methylation profile of the primarily diagnosed oligodendroglioma was available, six more patients were known to have a glioma prior to the development of an oligosarcoma. Ten patients received radio- and/or chemotherapy before developing an oligosaroma, nine patients did not get any of those treatments after resection of their prior glioma. Interestingly, the time span between primary diagnosis of an oligodendroglioma and manifestation of an oligosarcoma was highly variable and ranged from 1.2 to 32 years (Fig. 7a). Of note, for three patients the oligosarcoma was the first manifestation of a brain tumor (Table 1, Supplementary tables, online resource). Survival data for 17 patients were available.

The median overall survival of patients in which an oligosarcoma developed during the course of disease was about 11 years (Fig. 7b). Given that the timepoint at which the oligosarcoma occurred was highly variable and thereby its potential impact on survival, we focused on the clinical course since manifestation of oligosarcoma. The median overall survival after occurrence of oligosarcoma was 2.5 years. Since most of our cases occurred as a first recurrence, we determined the median survival for patients in which oligosarcoma occurred as first recurrence to be 1.8 years. Compared with recent data from the literature which report the median OS of IDH-mutant gliomas following a first episode of disease progression to be 8.3 years, this appeared clearly shortened, especially considering that most of the cases in this study were astrocytomas [24]. To corroborate this, we complied a cohort of patients from Heidelberg with molecularly defined oligodendroglioma in which a second operation took place at first recurrence and the diagnosis of conventional grade 3 oligodendroglioma was verified by histology and DNA-methylation profiling (Supplementary tables, online resource). In Kaplan–Meier analyses survival of patients in which oligosarcoma developed as first recurrence was highly significantly poorer as for patients with conventional grade 3 oligodendroglioma as first recurrence (*p* = 0.0038; Fig. 7c). There were survival data for three oligosarcoma cases in which no precursor lesion was known. All three patients died with similar survival times of 2.7, 3.3 and 3.7 years.

## Discussion

In this study we demonstrate that oligosarcomas are a distinct group of IDH-mutant glioma because it differs from conventional CNS WHO grade 3 oligodendrogliomas on multiple levels.

CNS WHO grade 2 and grade 3 oligodendrogliomas share a common DNA methylation profile, whereas both supratentorial as well as infratentorial IDH-mutant astrocytomas split into high-grade and low-grade methylation groups [6]. Primary mismatch repair deficient IDH-mutant astrocytomas (PMMRDIA) form another specific epigenetic group of IDH-mutant tumors [40]. Oligosarcomas display a unique DNA methylation profile, clearly separating them from all other tumor types, including other IDH-mutant gliomas. High-grade astrocytoma methylation groups as well as PMMRDIA show an attenuated CpG island methylator (G-CIMP)-phenotype, otherwise characteristic for all IDH-mutant gliomas. Interestingly, the G-CIMP-phenotype is attenuated in oligosarcomas as well, suggesting that reduced CpG methylation associates with a more aggressive biological behavior in all types of IDH-mutant gliomas. Recent findings revealed oligodendroglioma cells to have reduced global H3K27me3 levels compared to IDH-mutant astrocytomas, suggesting that 1p/19q codeletion interferes with epiproteomic modifications [17, 18, 28]. Surprisingly, H3K27me3 was abundantly present in oligosarcomas providing further evidence for a different epigenetic state of oligosarcomas compared to conventional oligodendrogliomas. Interestingly, both loss and increase of global H3K27me3 have been associated with malignant progression in different tumor types [22, 21, 26].

Given the importance of 1p/19q codeletion in oligodendroglioma diagnostics, the finding of five tumors in our cohort with intact 1p/19q or incomplete deletion is remarkable. All of these tumors arose from oligodendroglioma and in four of these a DNA methylation derived CNP was available showing complete 1p/19q codeletion in the primary tumor. For three of these samples SNP-array analyses could be performed demonstrating CN-LOH suggesting duplication of the retained chromosomal arm. Hiniker et al. described the same finding in a case of a 1p/19q-codeleted oligodendroglioma recurring as oligosarcoma without 1p/19q codeletion in FISH but copy number neutral LOH of 1p and 19q visible in SNP arrays data [19]. SNP array data also showed frequent occurrence of polyploidy in oligosarcoma which was not present in primary tumors. Interestingly, polysomy of 1p and 19q has been suggested as a negative prognostic factor in oligodendroglioma [13, 31].

Another molecular alteration suggested as driver of inferior survival in IDH-mutant gliomas and frequently present in oligosarcomas are homozygous *CDKN2A/B* deletions. According to cIMPACT-NOW update 5 and the upcoming WHO classification for tumors of the central nervous system, homozygous *CDKN2A/B* deletions correspond to WHO CNS grade 4 in IDH-mutant astrocytomas [23, 8]. The prognostic relevance of *CDKN2A/B* deletions for patients with genetically defined oligodendrogliomas is currently less well established. While two studies failed to demonstrate any prognostic relevance [3, 30], a recent study demonstrated a worse outcome for the small fraction of 7% of grade 3 oligodendrogliomas with homozygous *CDKN2A* deletions [4]. It is unknown whether or not tumors with sarcomatous features were present in these studies. In future studies, however, the existence of oligosarcomas as a distinct group of IDH-mutant tumors with frequent deletions of 1p/19q as well as *CDKN2A/B* should be considered in order to exclude an admixture with conventional oligodendrogliomas.

Immunohistochemical accumulation of p53 is often associated with pathogenic *TP53* mutations. In the context of an IDH-mutant glioma nuclear accumulation of p53 is commonly interpreted as indication of a mutation and thus as a marker of a non-1p/19q-codeleted IDH-mutant glioma [23]. In 11 of the 12 oligosarcomas examined, strong nuclear expression of p53 was found. We could indeed detect 2/12 oligosarcomas harboring pathogenic *TP53* mutations. *TP53* mutations have been detected in recurrent oligodendrogliomas in previous studies and cytotoxic treatments especially temozolomide are discussed to play a causative role [2, 7, 25]. However, only one oligosarcoma in our series carried missense mutations (K132N, G112S) expected to result in nuclear accumulation of p53, whereas the other one displayed a protein truncating stop-gain mutation. The very high frequency of p53 accumulation in oligosarcomas is therefore not sufficiently explained by *TP53* mutations alone.

Whole proteome analyses demonstrated a pronounced divergence of the proteome of oligosarcomas from that of conventional WHO CNS grade 3 oligodendrogliomas. About 30% of all quantified proteins are differentially abundant, a fraction about six times greater than that of oligodendroglioma versus astrocytoma (~ 5%; data not shown). Interestingly, upregulation of several marker proteins indicates smooth muscle differentiation to occur recurrently in oligosarcoma as reported in earlier studies [34, 41, 42]. Proteomic analyses also revealed downregulation of tumor suppressor NF1 in oligosarcoma. Interestingly, NF1 is frequently inactivated in mesenchymal glioblastoma, IDH-wildtype and gliosarcoma, IDH-wildtype [9, 46], suggesting that loss of NF1 may support glial-to-mesenchymal transition in general. While genetic inactivation of *NF1* either by deletion and/or mutation is detectable in oligosarcoma, the mechanism for aberrant overexpression of YAP1 is less clear. Notably, we found one oligosarcoma with a *YAP1* amplification as likely mechanism for overexpression. However, in the other cases, YAP1 overexpression was not associated with mutations, amplifications or fusions of the *YAP1* gene indicating that other mechanisms remain to be determined. Of note, YAP1 may be of interest as a therapeutic target because pharmacologic inhibition of YAP/TAZ dependent transcription has shown efficiency in pre-clinical studies of different cancer types including glioblastoma [15, 45].

The data show that oligosarcomas were diagnosed as primary tumors or as recurrences from conventional lower grade oligodendrogliomas. This suggests that oligosarcomas may either develop as a distinct progression variant from oligodendroglioma or arise de novo. Throughout all our analyses we did not find any differences between primary tumors of oligosarcomas and conventional oligodendrogliomas, which indicates that all oligodendrogliomas might harbor the potential to progress to oligosarcoma. Of note, recently an oligodendroglioma which acquired an imbalanced 1p/19q codeletion and a *TP53* mutation at recurrence without histological signs of oligosarcomatous transformation was reported, possibly representing an intermediate state in the course of oligosarcoma development [25]. Occurrence of oligosarcoma in several treatment-naïve individuals demonstrates that cytotoxic treatments are not necessary for their development.

For the comparative assessment of oligosarcoma prognosis the general prognosis and the specific prognosis after the first recurrence of oligodendroglioma, IDH-mutant and 1p/19q codeleted appears important. Historic studies are not helpful due to the usual heavy admixture of 1p/19q-wt and IDH-wt tumors in these cohorts. Nowadays, the median overall survival of molecular defined oligodendrogliomas, IDH-mutant and 1p/19q codeleted can be estimated to be in the range of 15–20 years, if treated with radiochemotherapy [10, 43]. In fact, there is no single study available in which the median survival for molecularly defined oligodendroglioma grade 2 has been reached, which was the first manifestation of a brain tumor in the majority of our oligosarcoma cohort. The oligodendroglioma-specific median survival after the first progression is currently unknown. However, a recent study reported a median overall survival of 8.3 years after the first progression of IDH-mutant gliomas [24]. Of note, about 2/3 of cases in this study were astrocytomas which are expected to have a less favorable outcome than oligodendrogliomas. A median survival time of 1.8 years for patients with oligosarcomas as first recurrence, therefore, favors a poorer prognosis. The additional three primary oligosarcoma patients with survival time less than 4 years argues for a more aggressive biological behavior of oligosarcomas compared to conventional oligodendrogliomas.

Since these retrospective analyses have several limitations like potential unintended selection bias, heterogeneity of treatment protocols and a non-uniform clinical observation, more data in future studies will be necessary for confirmation.

It will be a matter of debate whether oligosarcoma should be recognized as a distinct tumor type or rather as a distinct subtype in the overarching tumor type 'oligodendroglioma, IDH-mutant and 1p/19q-codeleted'. The interpretation that oligosarcoma is a distinct subtype of oligodendroglioma is supported by the fact that it often develops from a conventional oligodendroglioma, preserves the usual mutation signature and the 1p/19q LOH remains detectable even in the absence of a copy number alteration. A subtype definition would more clearly emphasize the clonal relationship between oligosarcoma and conventional oligodendroglioma and thus support a pathogenetic approach of classification.

In contrast, the perception of oligosarcoma as a separate tumor type is supported by the distinct phenotype and the considerable epigenetic and proteomic differences compared to oligodendroglioma. These differences between oligosarcomas and oligodendrogliomas are of a magnitude more commonly observed between different tumor types than between subtypes or different tumor grades of a tumor type. The process of malignant progression from oligodendroglioma to oligosarcoma is, therefore, somewhat reminiscent of the sarcomatous transformation of neurofibroma to malignant peripheral nerve sheath tumor, the latter of which is clearly recognized as a distinct entity by WHO.

Another question to be answered is that of an appropriate grade for oligosarcomas. Provided that the available clinical data will be confirmed in future studies a grade 4 designation should be considered as the clinical course appears more aggressive than that of conventional grade 3 oligodendrogliomas.

Irrespective of the precise taxonomy of oligosarcomas, it can be expected that the identification of oligosarcomas as a distinct group within the family of IDH-mutant gliomas will facilitate their diagnosis in the future and will help to further elucidate the clinical and molecular characteristics of these tumors. The diagnosis of oligosarcoma can be established by the combination of an IDH-mutant tumor with at least partial (leiomyo)sarcomatous differentiation which harbors a 1p/19q LOH which may be copy number neutral and typically (but not always) a *TERT* promoter mutation. Additionally, it is helpful for diagnostics that oligosarcomas are often preceded by a canonical oligodendroglioma. The unique DNA methylation profile of oligosarcomas will be included in version 12 of the brain tumor classifier and can be used to make the diagnosis in unresolved cases.

## Supplementary Information

Below is the link to the electronic supplementary material.

Supplementary file1 (PDF 1598 KB)

Supplementary file2 (XLSX 40 KB)
