# Supplementary material for: Oligosarcomas, IDH-mutant are distinct and aggressive
Source: Acta Neuropathol. 2021 Dec 30;143(2):263–81. doi: 10.1007/s00401-021-02395-z (PMC8742817; doi:10.1007/s00401-021-02395-z)
Supplement: Supplementary file 1 — Supplementary file1 (PDF 1598 KB) [file 401_2021_2395_MOESM1_ESM.pdf]

Supplementary Figure 1

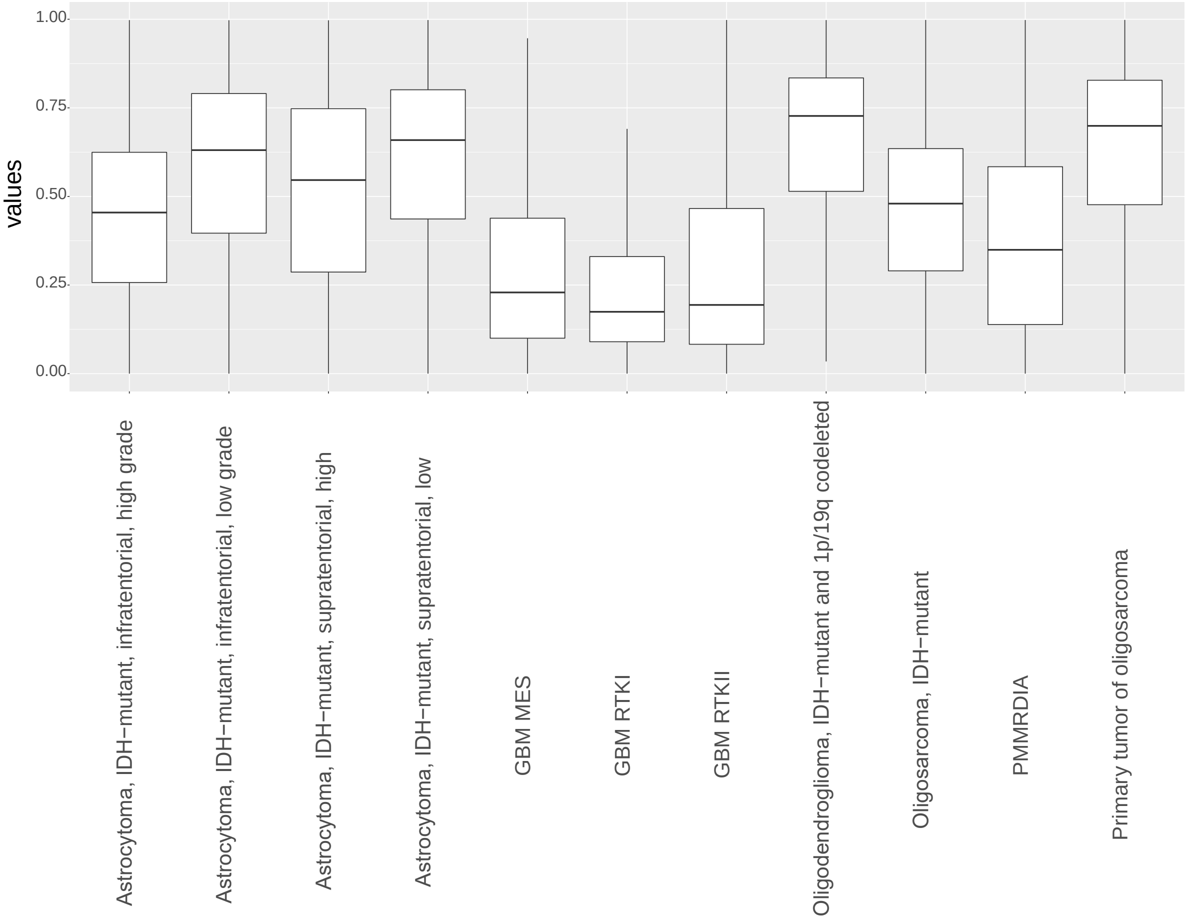

Mean DNA-methylation values of the 20000 most differentially methylated positions among different types of gliomas.
Beta-values vary from 0 (unmethylated) to 1 (fully methylated).

Supplementary Figure 2

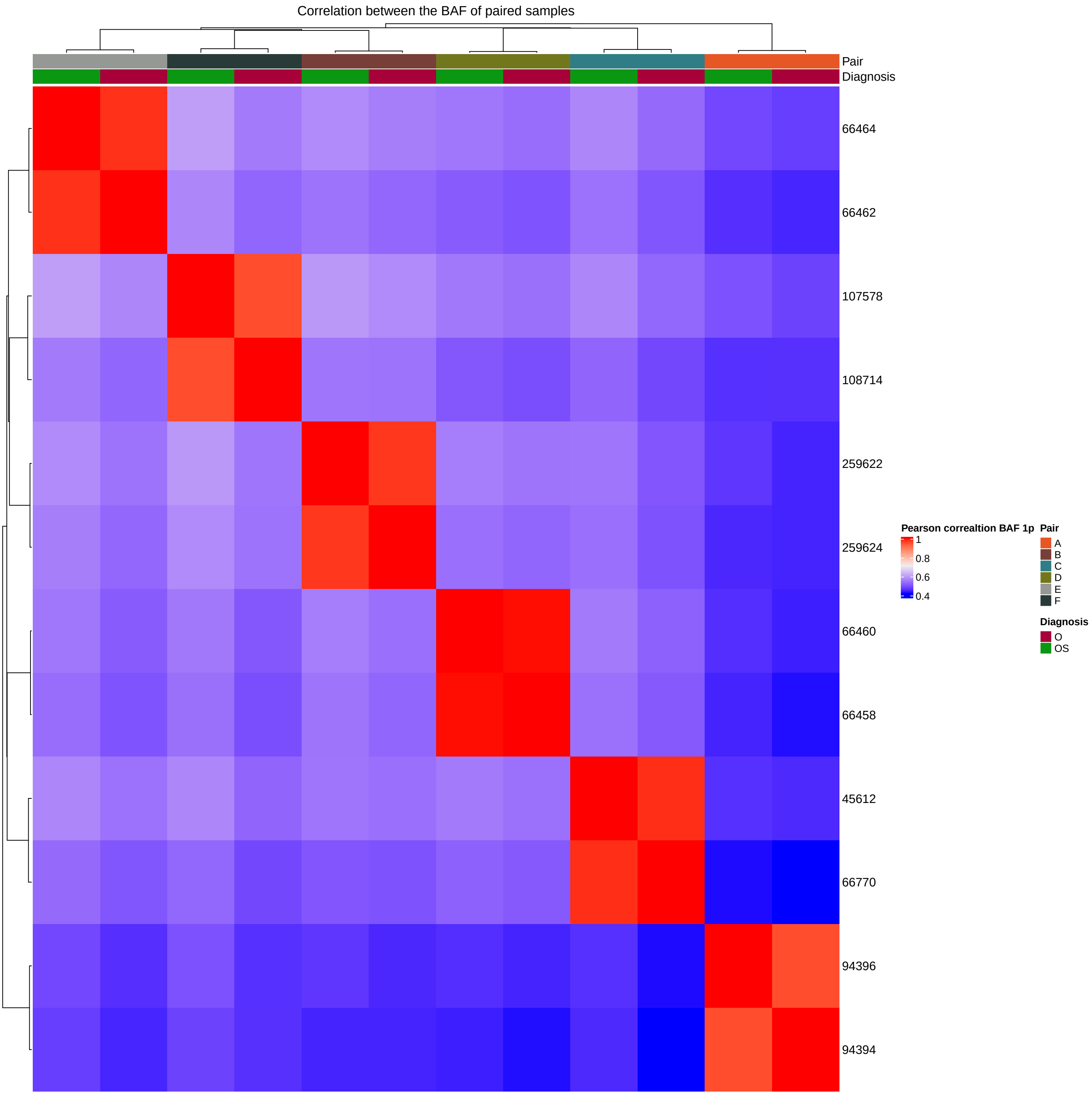

Supplementary Figure 3

Patient 1

Primary tumor

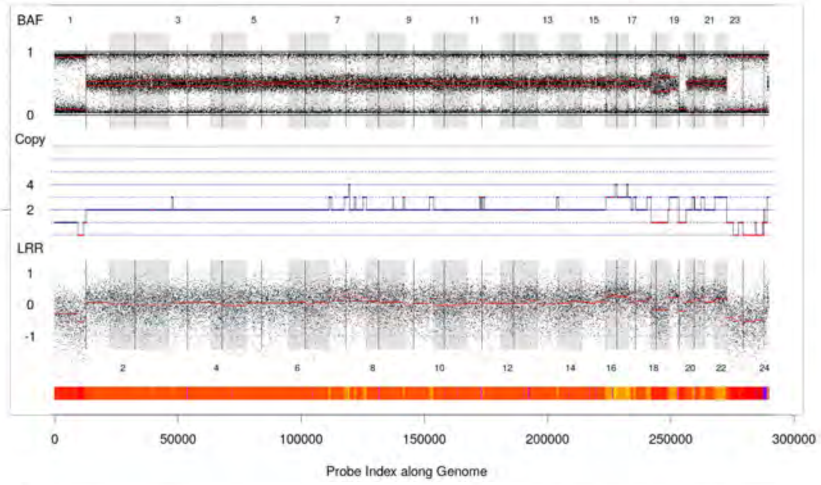

Summary and Centromeres

Break counts >50 = 88
Chromosome counts = 48
Centromere counts = 87
DNA index = 0.96
Ploidy detected = 2
% base state = 75
% '1' copy loss = 8
% accuired homo = 16

Chr	Copy	B allele	SNPs	Chr	Copy	B allele	SNPs
1 p	1	1	1018	12 p	2	1	4790
1 q	2	1	9765	12 q	2	2	281
2 p	2	1	10133	13 q	2	1	8008
2 q	2	1	6312	14 q	2	1	4160
3 p	2	0	252	15 q	2	1	3121
3 q	2	0	186	16 p	4	4	104
4 p	2	1	4518	16 q	3	2	1069
4 q	2	1	3841	17 p	3	2	810
5 p	2	1	6352	17 q	3	2	236
5 q	2	1	11109	18 p	1	1	2043
6 p	2	1	6572	18 q	1	1	4792
6 q	2	1	2958	19 p	3	2	184
7 p	3	2	576	19 q	1	1	2939
7 q	3	2	1070	20 p	3	2	938
8 p	2	1	4849	20 q	2	1	138
8 q	2	0	364	22 q	3	2	4826
9 p	2	1	3736	23 p	1	1	1847
9 q	2	1	6366	23 q	0	0	5109
10 p	2	1	4241	24 p	2	0	351
10 q	2	1	415	24 q	1	0	833
11 p	3	0	343				
11 q	2	1	548				

Oligosarcoma

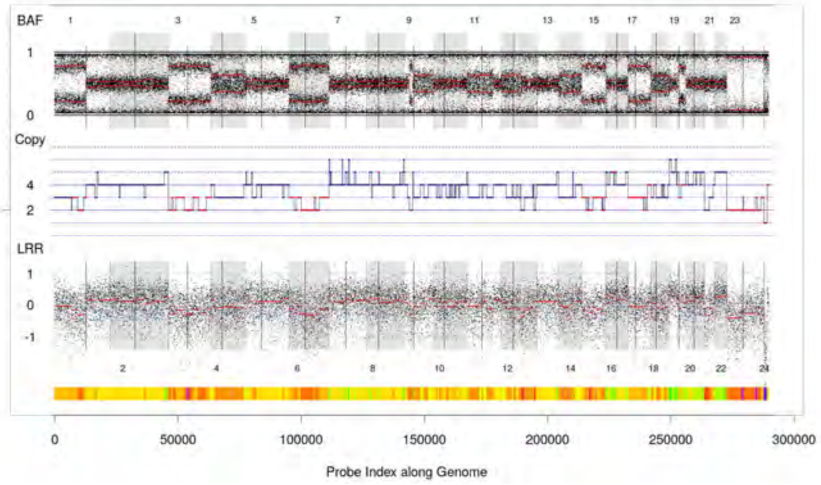

Summary and Centromeres

Break counts >50 = 190
Chromosome counts = 81.5
Centromere counts = 147
DNA index = 1.71
Ploidy detected = 4
% base state = 33
% '1' copy loss = 13
% accuired homo = 35

Chr	Copy	B allele	SNPs	Chr	Copy	B allele	SNPs
1 p	3	3	1014	12 p	4	3	512
1 q	4	2	3575	12 q	3	2	1313
2 p	4	2	6157	13 q	4	2	8008
2 q	4	2	3626	14 q	3	2	1784
3 p	2	0	252	15 q	4	4	144
3 q	2	0	186	16 p	4	4	104
4 p	3	2	3210	16 q	4	2	3585
4 q	3	2	3841	17 p	3	3	3023
5 p	3	2	243	17 q	4	4	57
5 q	4	2	8556	18 p	4	3	1030
6 p	2	2	1670	18 q	3	2	459
6 q	2	2	3623	19 p	5	3	184
7 p	5	4	129	19 q	3	3	958
7 q	5	3	590	20 p	5	3	397
8 p	4	2	2525	20 q	4	2	44
8 q	5	5	283	22 q	5	3	3563
9 p	5	3	418	23 p	2	0	452
9 q	3	2	2645	23 q	3	0	318
10 p	4	2	1046	24 p	1	0	351
10 q	4	2	768	24 q	1	0	834
11 p	4	4	343				
11 q	3	2	545				

Patient 3

Primary tumor

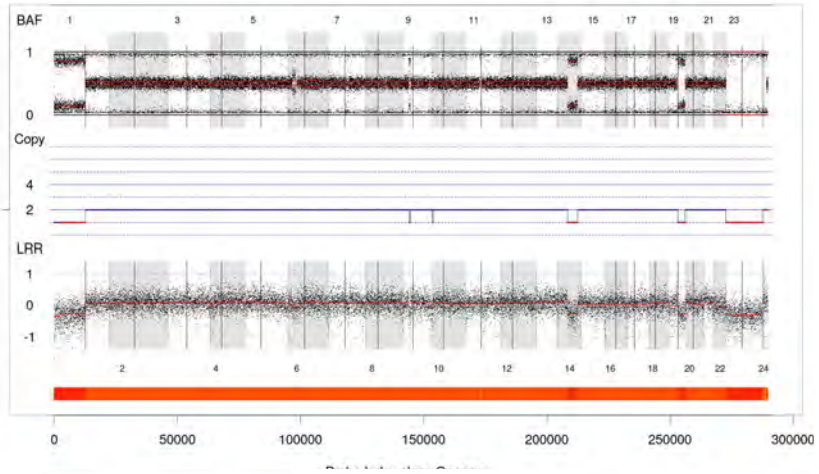

Summary and Centromeres

Break counts >50 = 48
Chromosome counts = 46
Centromere counts = 84
DNA index = 0.94
Ploidy detected = 2
% base state = 87
% '1' copy loss = 12
% accuired homo = 13

Chr	Copy	B allele	SNPs	Chr	Copy	B allele	SNPs
1 p	1	1	10377	12 p	2	1	4790
1 q	2	1	9031	12 q	2	1	9426
2 p	2	1	8016	13 q	2	1	8787
2 q	2	1	83	14 q	2	1	3965
3 p	2	1	7800	15 q	2	1	9792
3 q	2	1	9037	16 p	2	2	104
4 p	2	1	3077	16 q	2	1	4521
4 q	2	1	9653	17 p	2	1	3023
5 p	2	1	6352	17 q	2	1	597
5 q	2	1	11109	18 p	2	1	2043
6 p	2	1	3142	18 q	2	1	5401
6 q	2	1	5851	19 p	2	1	102
7 p	2	1	6814	19 q	1	1	2939
7 q	5	3	52	20 p	2	1	3324
8 p	2	1	4849	20 q	2	1	4126
8 q	2	2	225	22 q	2	1	4826
9 p	2	1	1043	23 p	1	1	6605
9 q	2	1	6540	23 q	1	1	8438
10 p	2	1	4241	24 p	2	2	351
10 q	2	1	7891	24 q	2	2	833
11 p	3	3	25				
11 q	2	1	7927				

Oligosarcoma

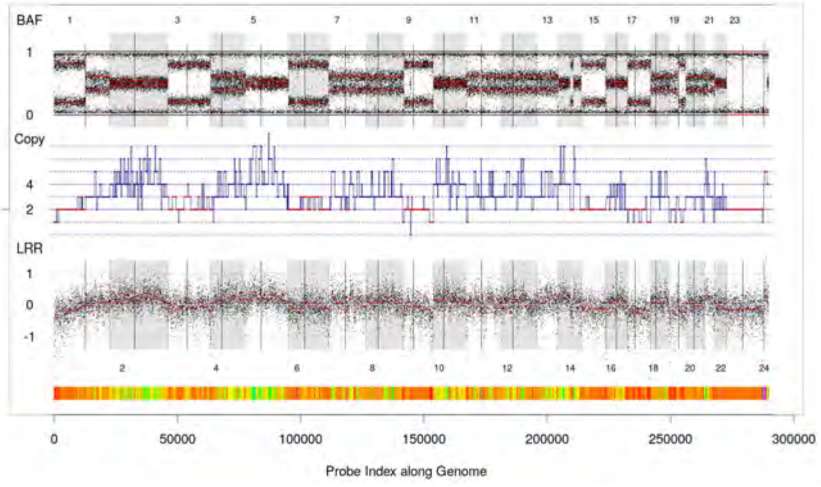

Summary and Centromeres

Break counts >50 = 500
Chromosome counts = 83
Centromere counts = 148
DNA index = 1.71
Ploidy detected = 4
% base state = 11
% '1' copy loss = 24
% accuired homo = 34

Chr	Copy	B allele	SNPs	Chr	Copy	B allele	SNPs
1 p	2	2	1161	12 p	2	1	16
1 q	3	2	3656	12 q	4	3	1432
2 p	4	2	349	13 q	3	2	498
2 q	3	0	189	14 q	6	3	74
3 p	3	3	1211	15 q	4	3	33
3 q	3	3	136	16 p	4	4	104
4 p	4	3	221	16 q	4	2	299
4 q	3	2	884	17 p	2	2	59
5 p	7	4	254	17 q	2	2	1537
5 q	6	3	81	18 p	5	5	59
6 p	3	3	521	18 q	3	2	1103
6 q	3	3	847	19 p	3	2	89
7 p	2	2	253	19 q	2	2	1021
7 q	6	3	59	20 p	3	2	3324
8 p	3	2	355	20 q	2	1	651
8 q	3	0	229	22 q	5	3	107
9 p	2	2	438	23 p	2	2	6605
9 q	2	2	3206	23 q	2	2	7975
10 p	4	4	109	24 p	5	5	72
10 q	4	2	102	24 q	5	0	823
11 p	6	6	98				
11 q	4	0	41				

Supplementary Figure 4

Patient 4

Primary tumor

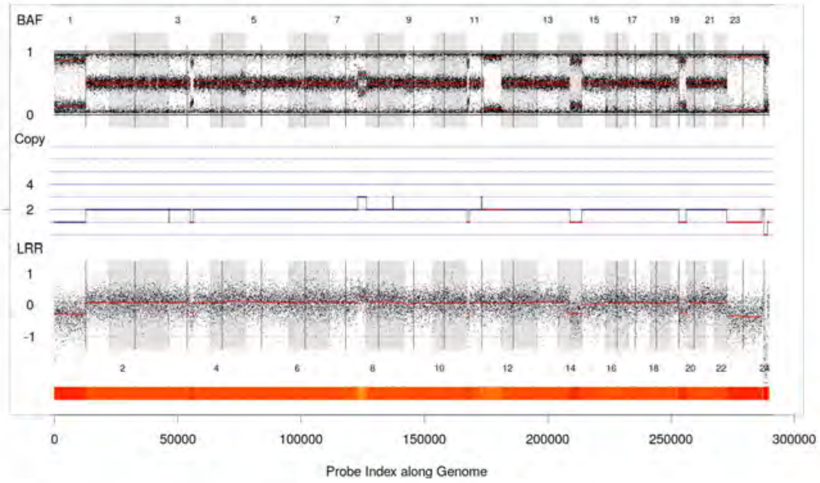

Summary and Centromeres

Break counts >50 = 63
Chromosome counts = 45
Centromere counts = 80
DNA index = 0.94

Ploidy detected = 2
% base state = 76
% '1' copy loss = 12
% accuired homo = 22

Chr	Copy	B allele	SNPs	Chr	Copy	B allele	SNPs
1 p	1	1	12779	12 p	2	1	4790
1 q	2	1	3237	12 q	2	1	896
2 p	2	1	307	13 q	2	1	8628
2 q	2	2	199	14 q	2	1	4575
3 p	2	2	88	15 q	4	2	28
3 q	2	1	147	16 p	2	1	3857
4 p	2	1	4518	16 q	2	2	114
4 q	2	1	9653	17 p	2	1	2603
5 p	2	1	6352	17 q	2	1	6393
5 q	2	1	11109	18 p	2	1	2043
6 p	2	1	3287	18 q	2	2	227
6 q	2	1	215	19 p	2	2	739
7 p	2	1	6803	19 q	1	1	2939
7 q	3	3	55	20 p	2	1	3324
8 p	2	1	4849	20 q	2	1	4126
8 q	2	1	3284	22 q	2	1	3282
9 p	2	1	3736	23 p	1	1	6605
9 q	2	1	8126	23 q	1	1	7608
10 p	2	1	4241	24 p	0	0	351
10 q	2	1	9219	24 q	0	0	834
11 p	3	3	292				
11 q	2	1	774				

Oligosarcoma

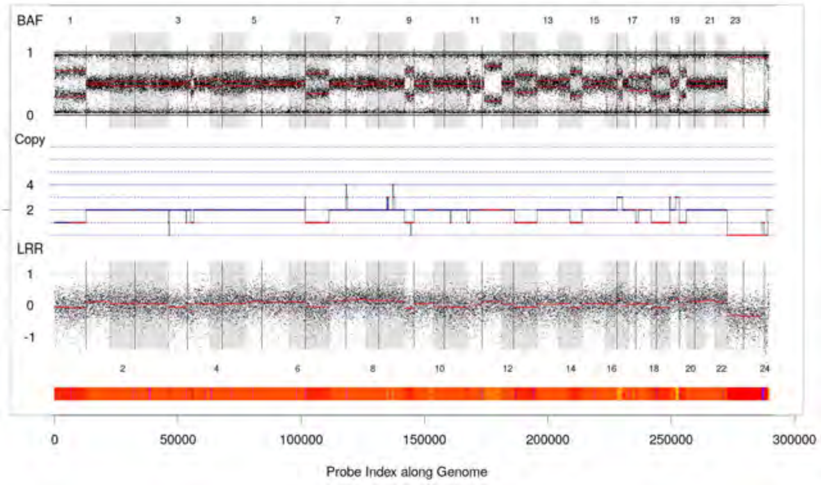

Summary and Centromeres

Break counts >50 = 96
Chromosome counts = 47
Centromere counts = 82
DNA index = 0.86

Ploidy detected = 2
% base state = 64
% '1' copy loss = 18
% accuired homo = 35

Chr	Copy	B allele	SNPs	Chr	Copy	B allele	SNPs
1 p	1	1	5759	12 p	2	1	1369
1 q	2	1	3237	12 q	2	1	369
2 p	2	1	307	13 q	2	1	8628
2 q	2	2	199	14 q	2	1	4575
3 p	2	2	88	15 q	6	6	25
3 q	2	1	1195	16 p	2	2	104
4 p	2	1	4071	16 q	3	2	2183
4 q	2	1	3845	17 p	2	2	831
5 p	2	1	3274	17 q	1	1	1130
5 q	2	1	11109	18 p	1	1	2043
6 p	2	1	28	18 q	1	0	255
6 q	1	1	215	19 p	5	0	16
7 p	2	1	6802	19 q	1	1	297
7 q	3	0	139	20 p	2	1	3324
8 p	2	1	4849	20 q	2	1	4126
8 q	2	1	3284	22 q	2	1	3282
9 p	1	1	1169	23 p	0	0	6605
9 q	2	1	6542	23 q	0	0	7606
10 p	2	1	4241	24 p	0	0	351
10 q	2	1	2406	24 q	0	0	834
11 p	6	5	67				
11 q	2	1	774				

Patient 11

Primary tumor

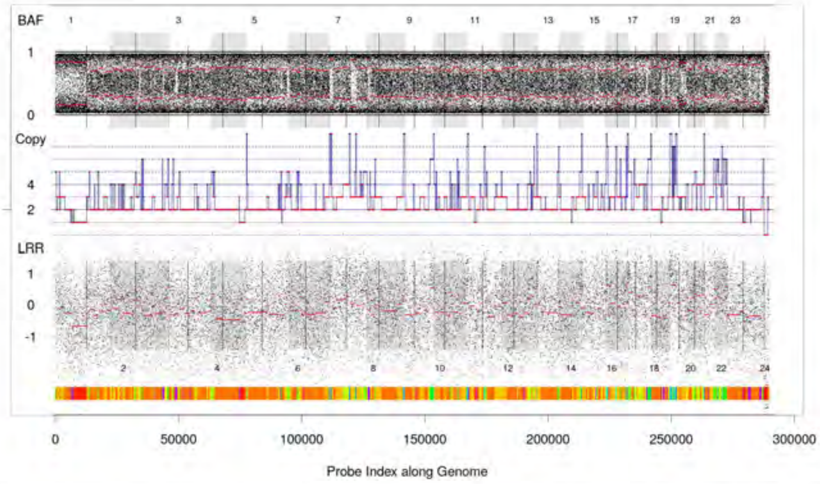

Summary and Centromeres

Break counts >50 = 368
Chromosome counts = 66
Centromere counts = 119
DNA index = 1.33

Ploidy detected = 4
% base state = 0
% '1' copy loss = 55
% accuired homo = 87

Chr	Copy	B allele	SNPs	Chr	Copy	B allele	SNPs
1 p	1	1	5083	12 p	4	0	26
1 q	3	3	165	12 q	2	2	1434
2 p	4	4	714	13 q	3	3	1472
2 q	5	5	220	14 q	3	3	528
3 p	2	2	1637	15 q	3	3	1336
3 q	2	2	870	16 p	3	3	152
4 p	2	2	3214	16 q	3	3	1229
4 q	2	2	6615	17 p	4	3	445
5 p	2	2	5761	17 q	7	4	30
5 q	2	2	1943	18 p	2	2	2043
6 p	2	2	795	18 q	2	2	1602
6 q	2	2	3929	19 p	3	3	855
7 p	4	4	1319	19 q	2	2	439
7 q	4	4	1159	20 p	4	4	932
8 p	3	3	1427	20 q	5	5	662
8 q	3	3	1049	22 q	4	4	418
9 p	4	3	404	23 p	2	1	23
9 q	2	2	571	23 q	3	0	133
10 p	3	3	161	24 p	0	0	351
10 q	3	3	782	24 q	0	0	1043
11 p	3	3	639				
11 q	2	2	576				

Oligosarcoma

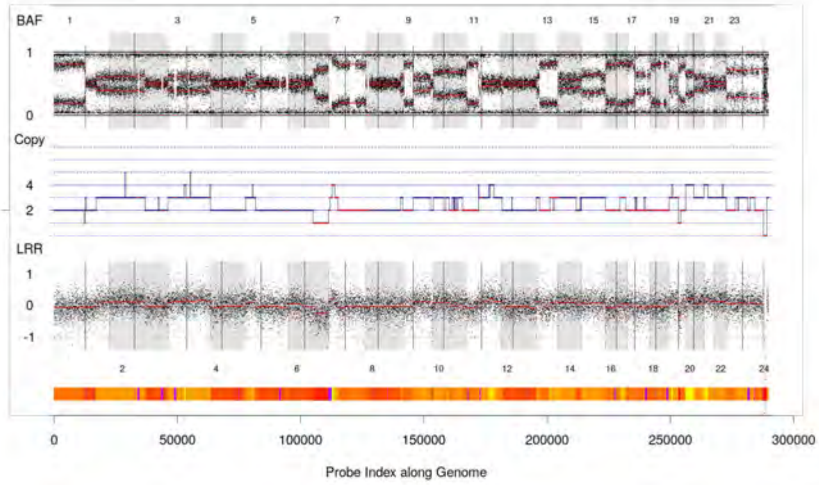

Summary and Centromeres

Break counts >50 = 150
Chromosome counts = 56.5
Centromere counts = 101
DNA index = 1.22

Ploidy detected = 2
% base state = 28
% '1' copy loss = 2
% accuired homo = 32

Chr	Copy	B allele	SNPs	Chr	Copy	B allele	SNPs
1 p	2	2	181	12 p	2	1	265
1 q	3	2	128	12 q	2	1	6334
2 p	3	2	3652	13 q	3	2	1281
2 q	3	2	272	14 q	3	2	7341
3 p	3	3	109	15 q	3	2	9792
3 q	3	2	493	16 p	2	2	175
4 p	2	1	4518	16 q	2	2	1471
4 q	2	1	5663	17 p	2	2	54
5 p	2	1	2231	17 q	3	2	403
5 q	2	1	7412	18 p	2	2	2043
6 p	2	1	1748	18 q	2	0	177
6 q	2	1	3418	19 p	3	3	299
7 p	2	2	2905	19 q	1	1	1002
7 q	2	2	8467	20 p	4	3	3324
8 p	2	1	1647	20 q	3	3	114
8 q	2	1	3762	22 q	3	3	107
9 p	2	2	1154	23 p	3	2	1716
9 q	3	2	7025	23 q	3	2	2058
10 p	3	2	4241	24 p	0	0	351
10 q	3	2	61	24 q	0	0	1043
11 p	4	0	22				
11 q	3	2	2708				

Supplementary Figure 5

Patient 13

Primary tumor

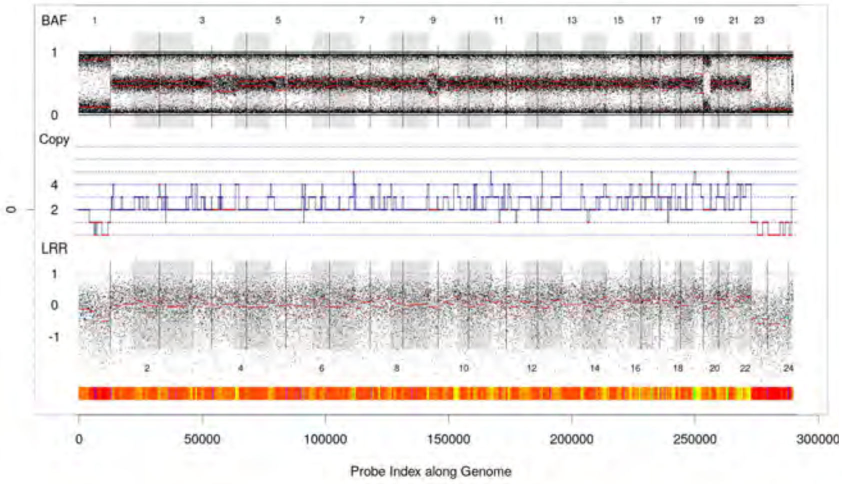

Summary and Centromeres

Break counts >50 = 275
Chromosome counts = 58.5
Centromere counts = 105
DNA index = 1.14
Ploidy detected = 2
% base state = 48
% '1' copy loss = 4
% accuired homo = 20

Chr	Copy	B allele	SNPs	Chr	Copy	B allele	SNPs
1 p	1	1	1011	12 p	2	1	2670
1 q	2	2	156	12 q	3	2	125
2 p	3	2	723	13 q	2	1	8008
2 q	4	4	222	14 q	3	2	713
3 p	3	3	149	15 q	3	2	995
3 q	2	1	247	16 p	4	4	454
4 p	2	1	3127	16 q	2	0	123
4 q	3	2	574	17 p	3	2	390
5 p	2	2	222	17 q	5	3	56
5 q	2	1	124	18 p	3	2	1047
6 p	2	1	815	18 q	2	2	122
6 q	2	1	3773	19 p	3	2	909
7 p	2	1	3740	19 q	2	2	1256
7 q	3	2	294	20 p	4	2	381
8 p	3	2	220	20 q	3	2	2287
8 q	3	2	152	22 q	4	4	419
9 p	3	2	404	23 p	1	1	1864
9 q	2	1	2141	23 q	1	0	317
10 p	3	2	505	24 p	1	0	351
10 q	3	2	768	24 q	0	0	834
11 p	2	2	580				
11 q	2	1	718				

Oligosarcoma

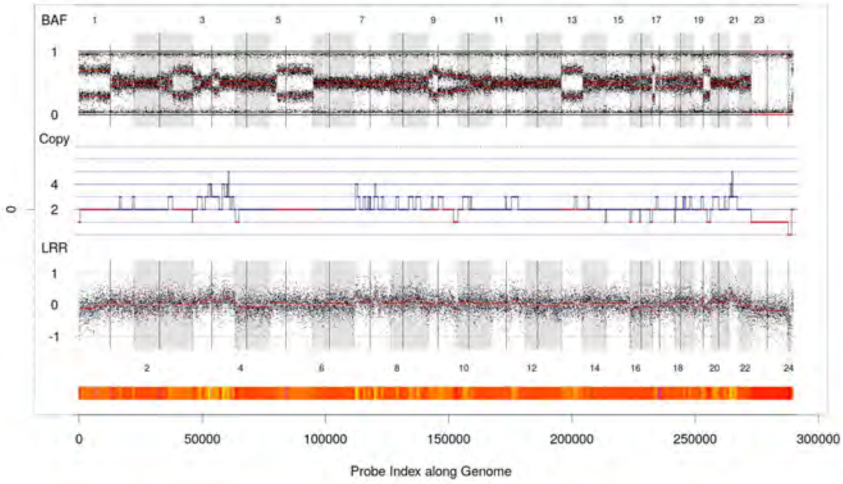

Summary and Centromeres

Break counts >50 = 156
Chromosome counts = 50.5
Centromere counts = 92
DNA index = 1.05
Ploidy detected = 2
% base state = 53
% '1' copy loss = 7
% accuired homo = 32

Chr	Copy	B allele	SNPs	Chr	Copy	B allele	SNPs
1 p	2	2	5385	12 p	2	1	4790
1 q	2	1	478	12 q	2	1	9456
2 p	2	1	10133	13 q	2	2	5444
2 q	2	0	283	14 q	2	1	2060
3 p	3	0	128	15 q	2	1	9792
3 q	3	2	2885	16 p	2	2	235
4 p	2	1	3137	16 q	2	1	3600
4 q	2	1	9653	17 p	2	1	442
5 p	2	2	219	17 q	2	1	188
5 q	2	2	202	18 p	4	2	53
6 p	2	1	1749	18 q	2	1	1005
6 q	2	1	6966	19 p	3	2	184
7 p	2	1	303	19 q	2	2	1585
7 q	6	3	42	20 p	3	2	2298
8 p	2	1	1794	20 q	2	1	661
8 q	2	1	2348	22 q	3	0	58
9 p	2	2	2079	23 p	1	1	6605
9 q	2	1	153	23 q	1	1	8438
10 p	3	2	2305	24 p	0	0	351
10 q	2	2	268	24 q	0	0	833
11 p	4	2	98				
11 q	2	1	1534				

Patient 23

Primary tumor

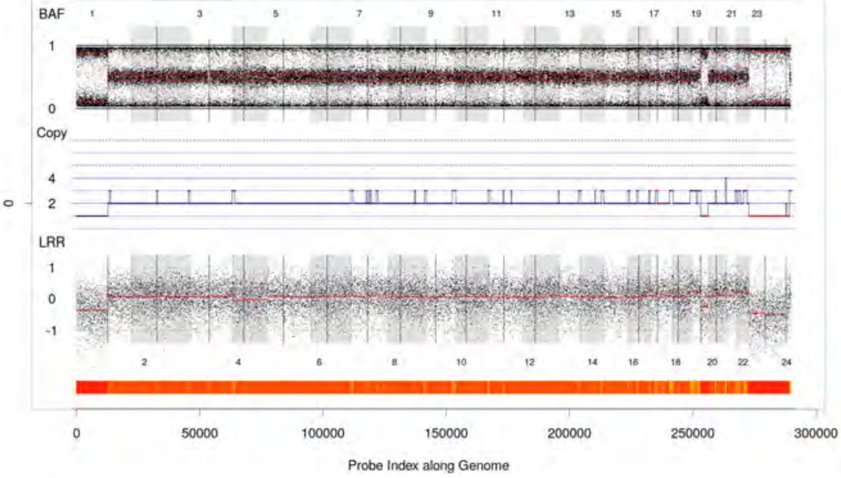

Summary and Centromeres

Break counts >50 = 119
Chromosome counts = 46
Centromere counts = 84
DNA index = 1
Ploidy detected = 2
% base state = 76
% '1' copy loss = 10
% accuired homo = 14

Chr	Copy	B allele	SNPs	Chr	Copy	B allele	SNPs
1 p	1	1	12779	12 p	2	2	164
1 q	2	2	84	12 q	2	2	100
2 p	2	1	5509	13 q	2	1	8049
2 q	3	2	272	14 q	2	1	4656
3 p	2	2	500	15 q	2	1	2530
3 q	2	2	124	16 p	2	2	143
4 p	2	1	3645	16 q	2	2	123
4 q	2	2	158	17 p	3	3	173
5 p	2	2	180	17 q	2	1	141
5 q	2	1	11109	18 p	2	2	111
6 p	2	2	118	18 q	2	2	517
6 q	2	1	9590	19 p	3	2	1223
7 p	2	1	370	19 q	1	1	2939
7 q	2	1	373	20 p	3	3	394
8 p	2	1	4849	20 q	2	1	37
8 q	2	2	267	22 q	2	1	179
9 p	2	1	3736	23 p	1	1	6605
9 q	2	1	6685	23 q	1	1	8438
10 p	2	2	147	24 p	2	2	351
10 q	2	2	213	24 q	1	1	833
11 p	3	3	294				
11 q	2	1	2967				

Oligosarcoma

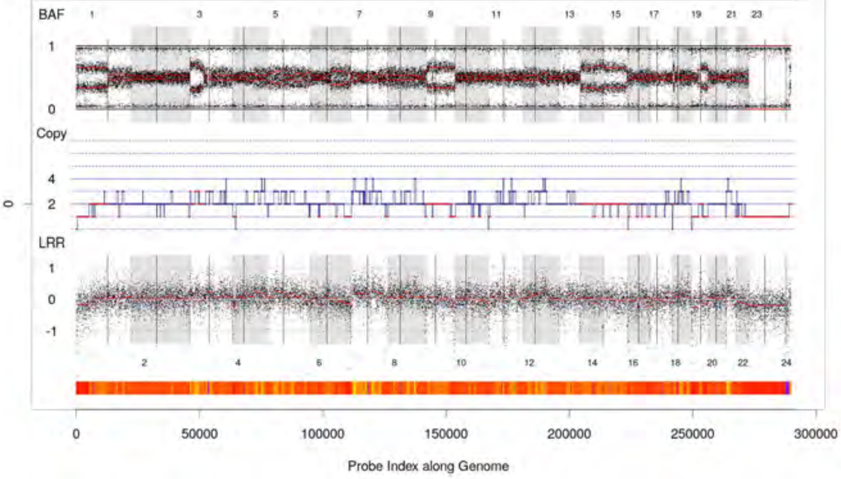

Summary and Centromeres

Break counts >50 = 339
Chromosome counts = 52
Centromere counts = 95
DNA index = 1.02
Ploidy detected = 2
% base state = 49
% '1' copy loss = 14
% accuired homo = 34

Chr	Copy	B allele	SNPs	Chr	Copy	B allele	SNPs
1 p	2	2	1161	12 p	3	2	238
1 q	2	1	2978	12 q	3	2	1433
2 p	2	1	3588	13 q	2	1	1754
2 q	1	1	189	14 q	2	2	5103
3 p	3	0	500	15 q	2	2	2605
3 q	2	1	136	16 p	2	1	199
4 p	2	1	3139	16 q	2	1	289
4 q	2	0	158	17 p	2	1	70
5 p	3	2	334	17 q	2	1	515
5 q	3	2	491	18 p	3	0	59
6 p	2	1	1738	18 q	3	0	165
6 q	3	2	785	19 p	2	2	734
7 p	2	1	302	19 q	2	2	592
7 q	6	4	55	20 p	2	1	119
8 p	2	1	1018	20 q	2	1	3781
8 q	2	0	200	22 q	3	2	107
9 p	2	2	539	23 p	1	1	6605
9 q	2	2	5288	23 q	1	1	8438
10 p	2	1	4241	24 p	1	0	351
10 q	2	0	102	24 q	1	0	833
11 p	4	4	292				
11 q	3	0	51				

Supplementary Figure 6

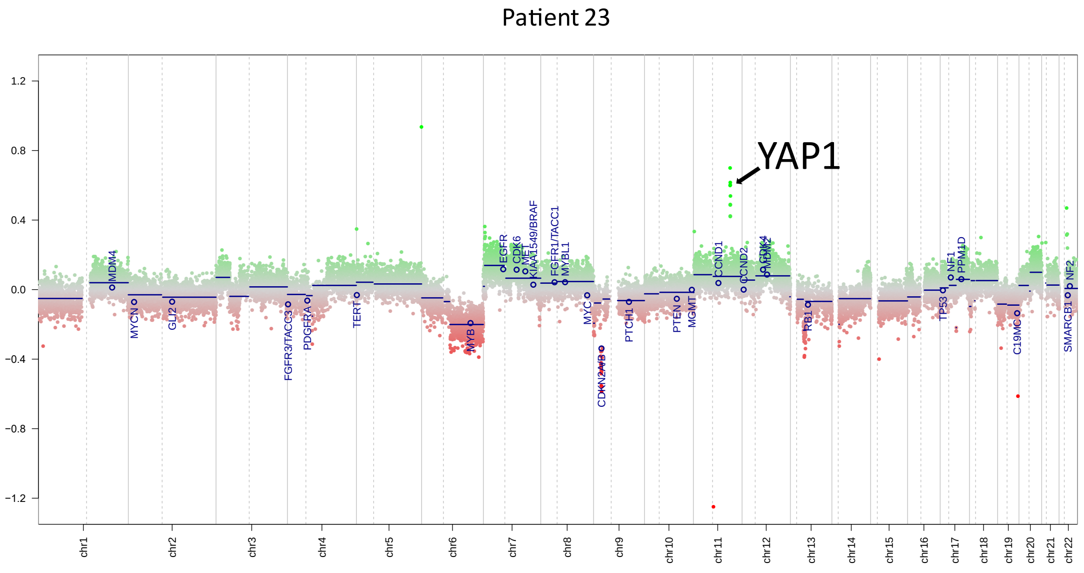

Supplementary Figure 7

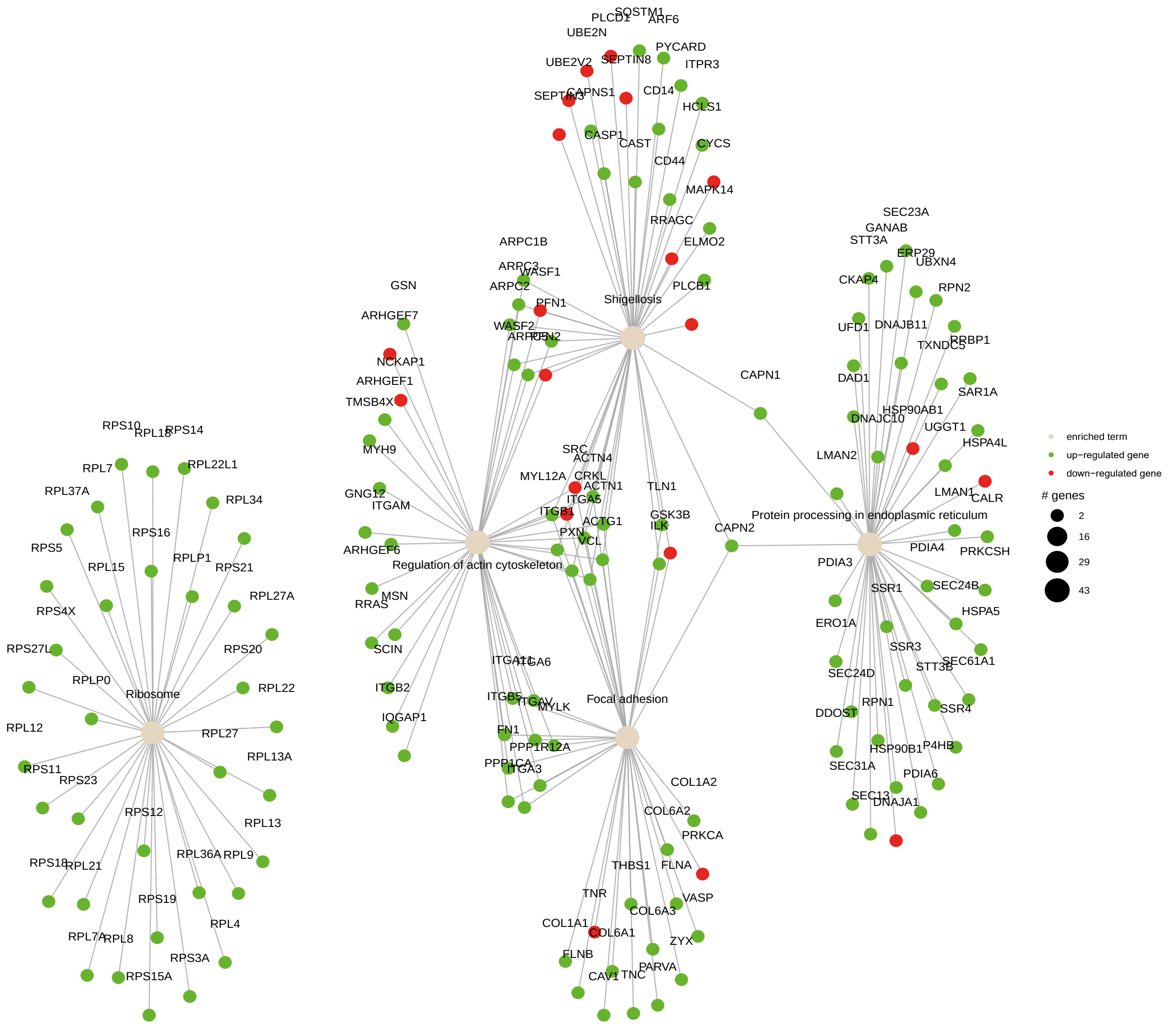

PathfindR analysis of the proteome of oligosarcoma versus oligodendroglioma grade 3. Dendrogram showing enriched pathways. Green marked proteins are upregulated and red marked proteins are down-regulated in oligosarcoma.

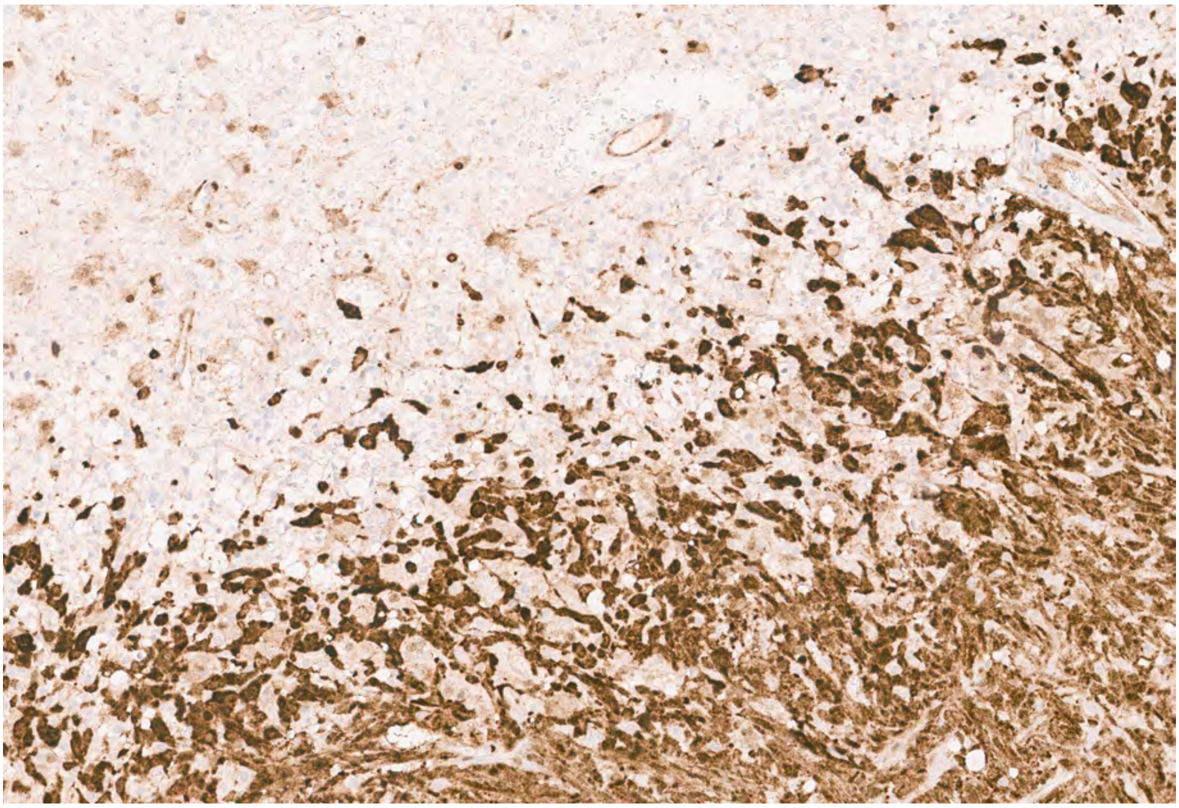

Immunohistochemistry shows strong expression of YAP1 in tumor cells of the oligosarcoma component but not in the oligodendroglioma component.
